# A degron-based strategy reveals new insights into Aurora B function in *C*. *elegans*

**DOI:** 10.1371/journal.pgen.1009567

**Published:** 2021-05-20

**Authors:** Nikita S. Divekar, Amanda C. Davis-Roca, Liangyu Zhang, Abby F. Dernburg, Sarah M. Wignall

**Affiliations:** 1 Department of Molecular Biosciences, Northwestern University, Evanston, Illinois, United States of America; 2 Department of Molecular and Cell Biology, University of California, Berkeley, California, United States of America; University of California Santa Cruz, UNITED STATES

## Abstract

The widely conserved kinase Aurora B regulates important events during cell division. Surprisingly, recent work has uncovered a few functions of Aurora-family kinases that do not require kinase activity. Thus, understanding this important class of cell cycle regulators will require strategies to distinguish kinase-dependent from independent functions. Here, we address this need in *C*. *elegans* by combining germline-specific, auxin-induced Aurora B (AIR-2) degradation with the transgenic expression of kinase-inactive AIR-2. Through this approach, we find that kinase activity is essential for AIR-2’s major meiotic functions and also for mitotic chromosome segregation. Moreover, our analysis revealed insight into the assembly of the ring complex (RC), a structure that is essential for chromosome congression in *C*. *elegans* oocytes. AIR-2 localizes to chromosomes and recruits other components to form the RC. However, we found that while kinase-dead AIR-2 could load onto chromosomes, other components were not recruited. This failure in RC assembly appeared to be due to a loss of RC SUMOylation, suggesting that there is crosstalk between SUMOylation and phosphorylation in building the RC and implicating AIR-2 in regulating the SUMO pathway in oocytes. Similar conditional depletion approaches may reveal new insights into other cell cycle regulators.

## Author summary

During cell division, chromosomes must be accurately partitioned to ensure the proper distribution of genetic material. In mitosis, chromosomes are duplicated once and then divided once, generating daughter cells with the same amount of genetic material as the original cell. Conversely, during meiosis chromosomes are duplicated once and divided twice, to cut the chromosome number in half to generate eggs and sperm. One important protein that is required for both mitotic and meiotic chromosome segregation is the kinase Aurora B, which phosphorylates a variety of other cell division proteins. However, previous research has shown that some kinases have functions that are independent of their ability to phosphorylate other proteins. Thus, fully understanding how Aurora B regulates cell division requires methods to test whether its various functions require kinase activity. We designed and implemented such a strategy in the model organism *C*. *elegans*, by depleting Aurora B from meiotically and mitotically-dividing cells, leaving in place a kinase-inactive version. This work has lent insight into how Aurora B regulates cell division in *C*. *elegans*, and also serves as a proof of principle for our approach, which can now be applied to study other essential cell division kinases.

## Introduction

The Aurora family of serine/threonine kinases mediates critical cell division events. Some eukaryotes express a single Aurora kinase (*e*.*g*., Ipl1 in *Saccharomyces cerevisiae*), while in other organisms this kinase has undergone duplication and divergence, leading to specialized functions of distinct family members. For example, Aurora A localizes to spindle poles and promotes centrosome maturation and spindle assembly in mitosis, while Aurora B (together with Aurora C, in mammalian meiosis) is part of the multisubunit chromosome passenger complex (CPC), which regulates chromosome segregation and cytokinesis (reviewed in [1]).

While Aurora kinases are known to phosphorylate numerous important cell division proteins, a few kinase-independent roles for these proteins have been recently documented. For example, studies in human cells have shown that the CPC protects centromeric cohesion independent of Aurora B’s kinase activity [2]. In *Xenopus* egg extracts, the CPC has a kinase-dependent role in phosphorylating outer kinetochore components and a kinase-independent role in ensuring the proper composition of inner kinetochore proteins [3]. Thus, understanding this important family of cell division regulators requires distinguishing between kinase-dependent and independent functions.

In *C*. *elegans*, Aurora A and B (AIR-1 and AIR-2, respectively) are known to regulate essential events during both mitosis and meiosis. AIR-1 plays several roles during mitosis, including in centrosome maturation and microtubule nucleation (reviewed in [4]), and also is required for spindle assembly in oocytes [5]. Similarly, AIR-2 has multiple functions. In mitotically-dividing embryos, depletion of AIR-2 by RNAi leads to defects in chromosome segregation, the formation of the anaphase spindle midzone, and cytokinesis [6–10]. Similarly, AIR-2 is required for the segregation of homologous chromosomes and polar body extrusion during Meiosis I [8,11,12]. AIR-2 is also essential for the assembly of the “ring complex” (RC), which encircles the center of each bivalent during Meiosis I (and the sister-chromatid interface during Meiosis II) [13–16]. The CPC is required for localization of all other known RC components [14,15], and chromosomes lacking RCs or with improperly patterned RCs show defects in chromosome congression [13,17]. Finally, AIR-2 depletion has been reported to cause spindle defects in *C*. *elegans* oocytes [6,8,14,15], although what part of the spindle assembly pathway is affected has not been investigated.

Notably, it has been shown that one of AIR-1’s mitotic functions does not require kinase activity [18]. However, whether AIR-2 also has kinase-independent functions has not been examined. A number of studies have analyzed worms containing a mutation in the AIR-2 kinase domain (*air-2(or207))* and have demonstrated that these worms exhibit severe mitotic defects reminiscent of the phenotypes seen following AIR-2 RNAi [9,11,19]. However, this mutant exhibits some kinase activity in oocytes [19] and has no documented meiotic defects at the restrictive temperature, suggesting that this temperature-sensitive mutation may be buffered in germline cells by the activity of chaperones or other mechanisms. Thus, new strategies are needed to test whether the meiotic functions of AIR-2 require kinase-activity.

Here, we use the auxin-inducible degradation system in combination with transgene expression to address the role of AIR-2 in the meiotic divisions. Our analysis indicates that, in contrast to AIR-1, kinase activity is necessary for the major functions of AIR-2. Moreover, our studies uncovered new insights into the assembly of the RC in oocytes, demonstrating that there is crosstalk between phosphorylation and SUMOylation that promotes the assembly of this structure, and implicating AIR-2 in regulation of the SUMO pathway. Thus, our studies have yielded important new insights into this essential mediator of chromosome division.

## Results

### Development of a degron-based strategy to inhibit AIR-2/Aurora B kinase activity in *C*. *elegans* oocytes

Worms depleted of AIR-2 by RNAi have been shown to have a variety of meiotic defects. To determine whether these functions require kinase activity, we wished to analyze worms in which AIR-2 is expressed but is kinase dead. A number of groups have previously characterized a temperature-sensitive strain, *air-2(or207)*, with a mutation in the kinase domain. However, at the restrictive temperature (which ranges from 20–25°C), this mutant exhibited mitotic, but not meiotic defects [9,11,19–21]. Consistent with these previous studies, we found that ring complex (RC) and spindle assembly both appear to occur normally in *air-2(or207)* oocytes at 25°C (S1 Fig). Moreover, we stained *air-2(or207*) oocytes with an antibody that recognizes Histone H3 Serine 10 phosphorylation (H3S10p), a direct target of Aurora B/AIR-2 activity [22–24] and we found that this staining persisted in *air-2(or207)* oocytes at 25°C (S1A Fig), similar to previous observations of this strain at 20°C [19]. Thus, AIR-2 appears to have kinase activity in the germ line even at the restrictive temperature in this mutant.

This motivated us to design an alternate approach to inactivate AIR-2. We used the auxin inducible degradation (AID) method, in which a protein of interest is tagged at the endogenous locus with a short 44 amino acid degron tag, and the *Arabidopsis* ubiquitin ligase TIR1 is expressed in the tissue where the protein of interest will be degraded. Addition of the plant hormone auxin mediates an interaction between TIR1 and the degron tag, and subsequently leads to the ubiquitination and degradation of the degron-tagged protein via the proteasome [25,26]. We inserted a degron and GFP coding sequence into the endogenous *air-2* gene (*degron*∷*GFP*∷*air-2*) in a strain expressing TIR1 in the germ line using the *sun-1* promoter [25]; this enabled auxin-inducible degradation of endogenous AIR-2 specifically in the germ cells of the worm. Note that throughout this manuscript, when we refer to “endogenous AIR-2”, we are referring to this tagged version of the protein, with the degron and GFP tags added at the endogenous locus.

**Fig 1:**
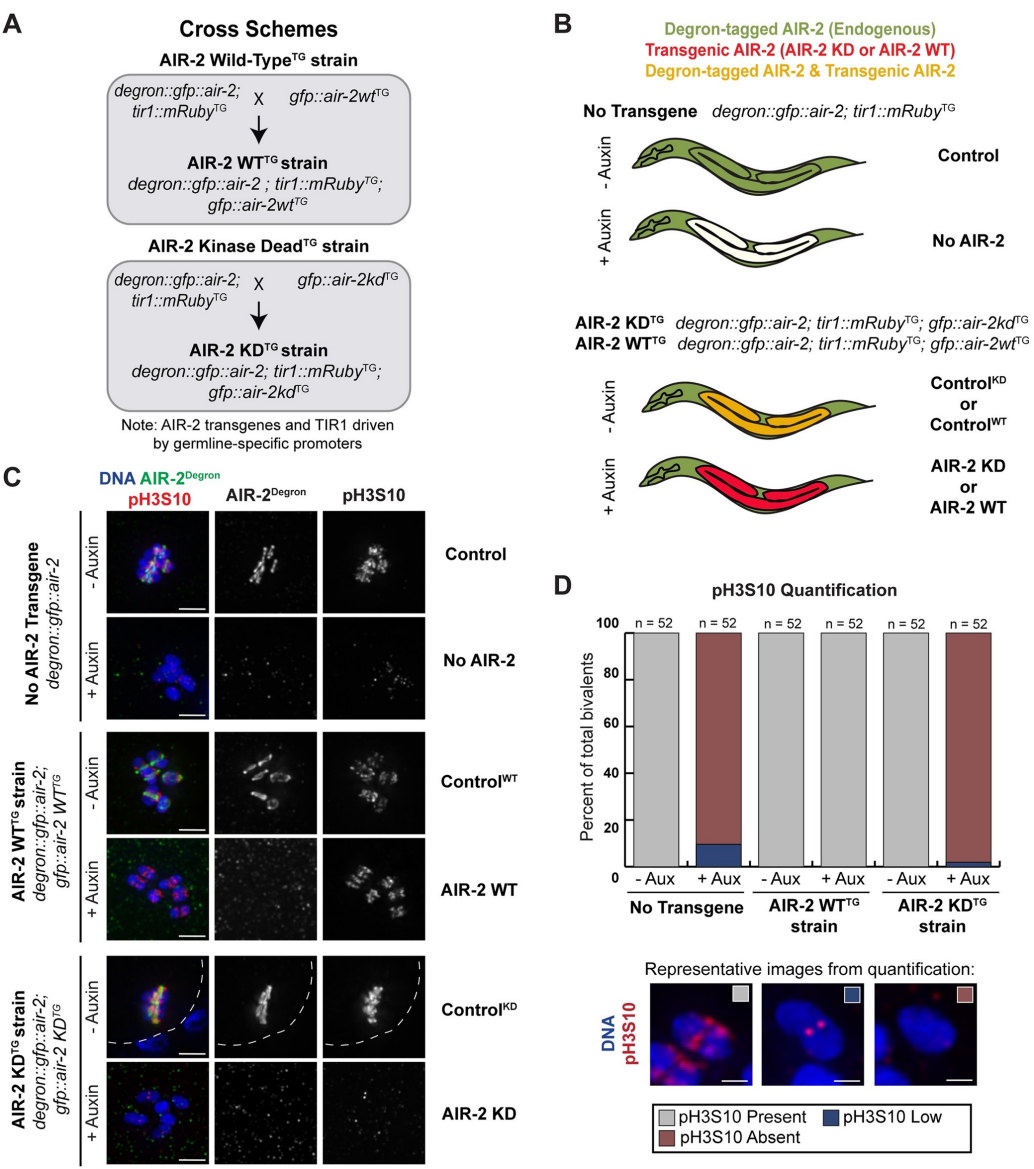
Design and validation of degron-based strategy to analyze Aurora B/AIR-2 kinase activity in *C*. *elegans* oocytes. (A) Cross-scheme for strain generation to study the kinase activity of AIR-2. A CRISPR-edited strain containing endogenous AIR-2 tagged with degron∷GFP and with TIR1 expressed in the germ line was crossed with a strain expressing transgenic GFP∷AIR-2 in the germ line. This resulted in a strain that expressed Degron∷GFP∷AIR-2, GFP∷AIR-2, and TIR1 in the germ line. To generate the AIR-2 KD experimental strain, the degron-tagged AIR-2 strain was crossed with a strain expressing kinase-dead GFP∷AIR-2 KD in the germ line. (B) Experimental scheme. Addition of auxin results in germline-specific depletion of endogenous AIR-2, leaving either no germline AIR-2 (“No AIR-2”), or a transgene-expressed version (“AIR-2 KD” or “AIR-2 WT”). (C) The degron antibody (green) was used to visualize endogenous AIR-2 and the H3S10 phosphorylation antibody (red) indicated the presence of active AIR-2; chromosomes shown in blue. In the absence of auxin, the degron antibody marks all six RCs and H3S10 phosphorylation is present on all six bivalents in each strain. Addition of auxin to the “No Transgene” and “AIR-2 KD^TG^” strains results in loss of H3S10 phosphorylation. However, H3S10 phosphorylation in the “AIR-2-WT^TG^” strain persists after auxin treatment. (D) Percent of bivalents that contain H3S10 phosphorylation marks in the different conditions, with representative images below (boxes in the corner of the images denote the category, description of categories in Materials and Methods). Bars = (C) 2.5μm; (D) 0.85μm.

We also introduced transgenes into this strain to express either wild-type AIR-2 (AIR-2 WT^TG^) or kinase-dead AIR-2 (AIR-2 KD^TG^); these transgenes were engineered using the *pie-1* promoter to drive germline expression (Fig 1A). In theory, exposure of these animals to auxin should result in depletion of degron-tagged endogenous AIR-2 in the germ line, leaving only the transgenic proteins (Fig 1B; the versions of AIR-2 expressed in the various conditions are summarized in S2A Fig). The AIR-2 KD transgene has a single amino acid mutation in the ATP-binding motif of the catalytic domain (K65M) [19] and is expressed at comparable levels to the AIR-2 WT transgene (S2B Fig), suggesting that this mutation does not cause folding defects that would cause the protein to be unstable. Furthermore, transgenic kinase-dead AIR-2 was previously shown to localize to the midbivalent in the presence of a wild-type version of AIR-2, supporting this view that the mutation does not alter the structure of the protein in a way that would affect its localization [19]. A kinase-dead form of human Aurora B with the corresponding lysine (K106) mutated has also been well characterized [27–30]; this mutant has been studied *in vitro* and has been stably expressed in cells, and is not thought to have major structural defects. Thus, depleting degron-tagged endogenous AIR-2 in worms expressing the K65M mutant version should allow us to analyze the contribution of kinase activity to the function of AIR-2. Although we cannot completely rule out the possibility that there are structural changes in the AIR-2 KD protein that could affect function in ways unrelated to the lack of kinase activity, our strategy nevertheless is a promising approach for exploring potential kinase-independent roles.

To validate this method, we first stained oocytes with an antibody that recognizes the degron tag to visualize the localization of endogenous AIR-2; we performed this analysis both in the original degron∷GFP∷AIR-2 strain lacking transgenes and also in strains with the AIR-2 WT and AIR-2 KD transgenes added. In the absence of auxin, the localization of tagged endogenous AIR-2 recapitulated previously-described AIR-2 enrichment at the central region of each bivalent (the “midbivalent”) in each of these strains, indicating that expression of the WT and KD AIR-2 transgenes does not affect the localization of the tagged endogenous protein (Fig 1C). We then transferred late larval stage (L4) worms onto auxin plates and incubated them until early adulthood to deplete the endogenously-expressed degron∷GFP∷AIR-2 from the germ line (via overnight incubation on auxin-containing plates) [26]. Upon auxin treatment, we observed that the anti-degron immunofluorescence was reduced to background levels in all three strains, demonstrating that degron-tagged endogenous AIR-2 was efficiently depleted from the oocytes, either substantially reducing AIR-2 in the germ line (Fig 1C, “No AIR-2”) or leaving one of the transgenic AIR-2 versions in place of the endogenous (Fig 1C; “AIR-2 WT” and “AIR-2 KD”). We confirmed this depletion by Western blotting of whole worms (S2B Fig) and also by quantifying GFP fluorescence in individual oocytes, as a means of assessing protein levels in the cells of interest (S2C and S2D Fig). The analysis of fluorescence intensity also revealed that the GFP∷AIR-2 KD transgene is expressed at higher levels in oocytes than the endogenously-expressed degron∷GFP∷AIR-2; the level of GFP fluorescence in the AIR-2 KD^TG^ strain following auxin incubation (representing only transgenic GFP∷AIR-2 KD) was higher than the fluorescence in the original degron∷GFP∷AIR-2 strain without auxin (representing tagged endogenous AIR-2) (S2C Fig). Moreover, transgenic GFP∷AIR-2 KD was also detected at higher levels than endogenously-expressed AIR-2 by Western blotting (S2B Fig; see Materials and methods for quantification). Thus, our data suggests that the AIR-2 KD transgene is slightly overexpressed relative to the level of endogenous AIR-2, but this expression does not affect the localization of the endogenous protein.

Next, we assessed H3S10 phosphorylation to evaluate AIR-2 kinase activity. H3S10 phosphorylation was detected on all six bivalents in the original degron∷GFP∷AIR-2 strain (Fig 1C and 1D “Control” condition) and was either low or was absent from the majority of bivalents in this strain in the presence of auxin (Fig 1C and 1D “No AIR-2” condition, see Materials and methods for quantification details for all Figures). Notably, the pH3S10 phospho-epitope was restored by expression of the wild-type AIR-2 transgene but was largely absent when the kinase-dead transgene was expressed (Fig 1C and 1D). Therefore, the kinase activity of AIR-2 is inhibited in the AIR-2 KD strain in the presence of auxin, allowing us to assess whether this activity is required for any of AIR-2’s meiotic functions.

### The kinase activity of AIR-2/Aurora B is required for proper CPC patterning in oocytes

**Fig 2:**
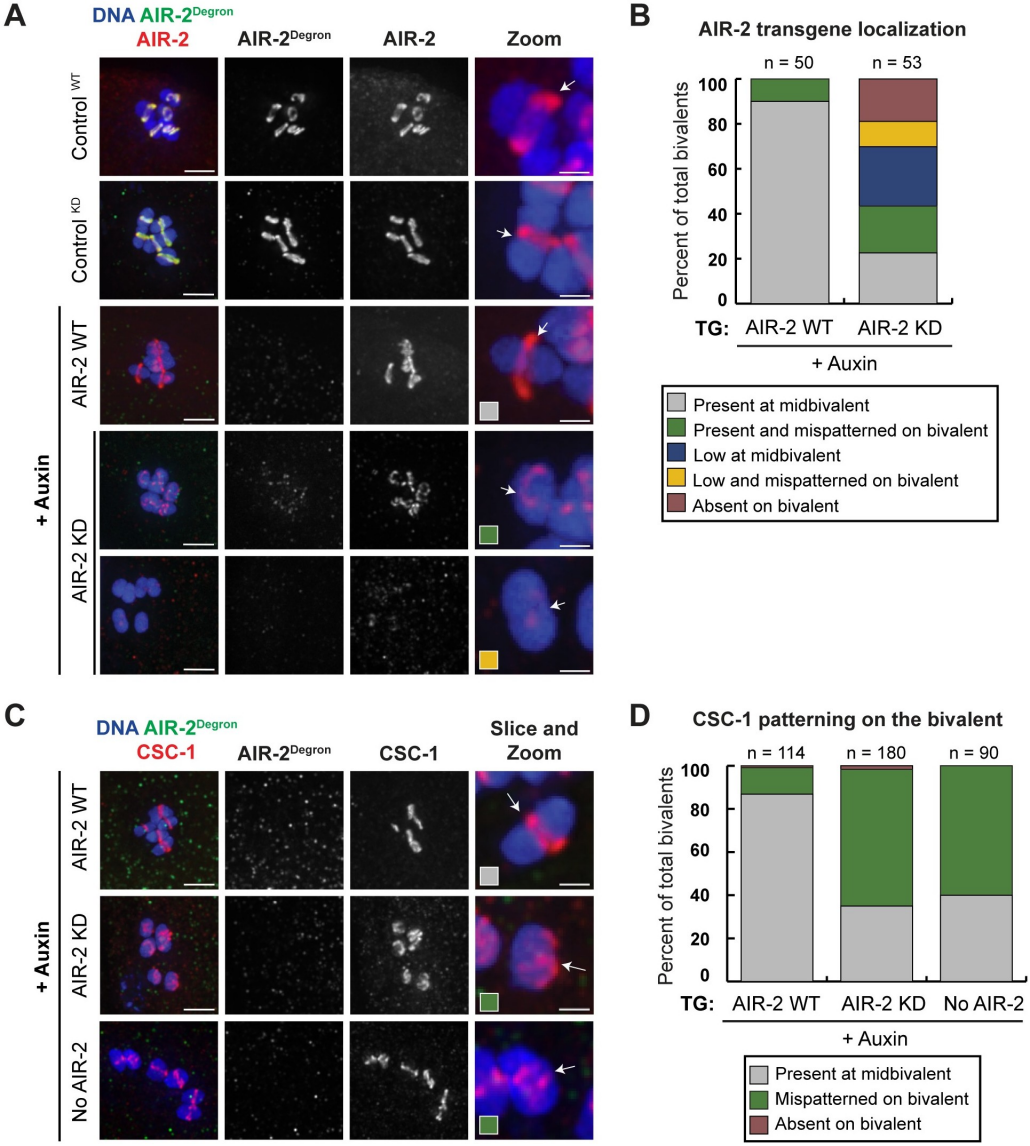
AIR-2/Aurora B kinase activity is required for proper Chromosomal Passenger Complex (CPC) recruitment and patterning on the bivalent. (A) In the absence of auxin (top two rows), transgenic wild-type and kinase-dead AIR-2 (visualized by the AIR-2 antibody, “AIR-2”, red) localize to the midbivalent when endogenous AIR-2 (visualized by the degron antibody, “AIR-2^Degron^”, green) is present. In the presence of auxin, endogenous AIR-2 is depleted; under these conditions the wild-type transgenic AIR-2 can localize to the midbivalent but the kinase-dead version is either mispatterned or absent. Arrows mark the midbivalent region and colored boxes in the zoomed images represent the categories quantified in part B. (B) Quantification of transgenic AIR-2 localization (either WT or KD) following depletion of endogenous AIR-2; descriptions of each category in Materials and methods. (C) All images are following auxin incubation, to deplete endogenous AIR-2 (imaged with the degron antibody, green). In the presence of wild-type transgenic AIR-2, CSC-1 (red) localizes to the midbivalent, while in the presence of transgenic kinase-dead AIR-2 or with no transgene, CSC-1 is mispatterned. Arrows mark the midbivalent region. (D) Quantification of CSC-1 localization following depletion of endogenous AIR-2, in strains expressing either transgenic WT or KD AIR-2, or with no transgenic AIR-2. Bars = (A) 2.5μm (A, zoom) 0.85μm; (C) 2.5μm (C, zoom) 0.85μm.

First, we tested whether the kinase activity of AIR-2 is required for its proper localization in oocytes. We used an AIR-2 antibody to visualize both endogenous and transgenic AIR-2 and the degron antibody to assess the degron-tagged endogenous version. Following auxin incubation to degrade endogenous AIR-2, we observed normal AIR-2 midbivalent localization in the strain containing the wild-type AIR-2 transgene (Fig 2A), suggesting that degradation of endogenous AIR-2 does not affect localization of the transgenic version. In contrast, kinase-dead AIR-2 did not localize properly when endogenous AIR-2 was degraded; AIR-2 KD^TG^ displayed normal midbivalent localization on only 26.6% of all chromosomes, and was either mispatterned (i.e., not at the midbivalent) (20.8%), low (26.4%), low and mispatterned (11.3%) or entirely absent (18.9%) from the rest (Fig 2B). We also conducted a shorter auxin incubation (4 hours on an auxin-containing plate) and noticed similar defects in the localization of the AIR-2 kinase-dead transgene (S3A Fig). Since most oocytes lacked detectable H3S10p signal (Fig 1D), while a much smaller fraction lacked AIR-2 KD staining (Fig 2B), the kinase activity of AIR-2 does not appear to be absolutely required for its localization to chromosomes. However, this activity does appear to be essential for AIR-2 to concentrate robustly at the midbivalent.

This finding led us to assess whether other CPC components require AIR-2 kinase activity for proper localization. Previous experiments in which AIR-2 was depleted using RNAi indicated that loading of the other CPC subunits (ICP-1, CSC-1, and BIR-1) is independent of AIR-2 [31]. In line with these results, we found that ICP-1, CSC-1, and BIR-1 localized to chromosomes in the absence of AIR-2 kinase activity. However, in the absence of AIR-2 kinase activity, these components were often mispatterned (Fig 2C and 2D, S3B, S3C and S3D Fig). Moreover, while there is normally a visible gap in the DNA staining between the two lobes of each bivalent, DAPI staining appeared contiguous in the absence of AIR-2 kinase activity (S4A Fig), similar to what was observed following *air-2(RNAi)* [32]. Overall, these results suggest that the kinase activity of AIR-2 is required for patterning the CPC on meiotic chromosomes and for generating normal bivalent structure.

### Kinase-dead AIR-2/Aurora B cannot serve as a scaffold for the loading of other RC components

**Fig 3:**
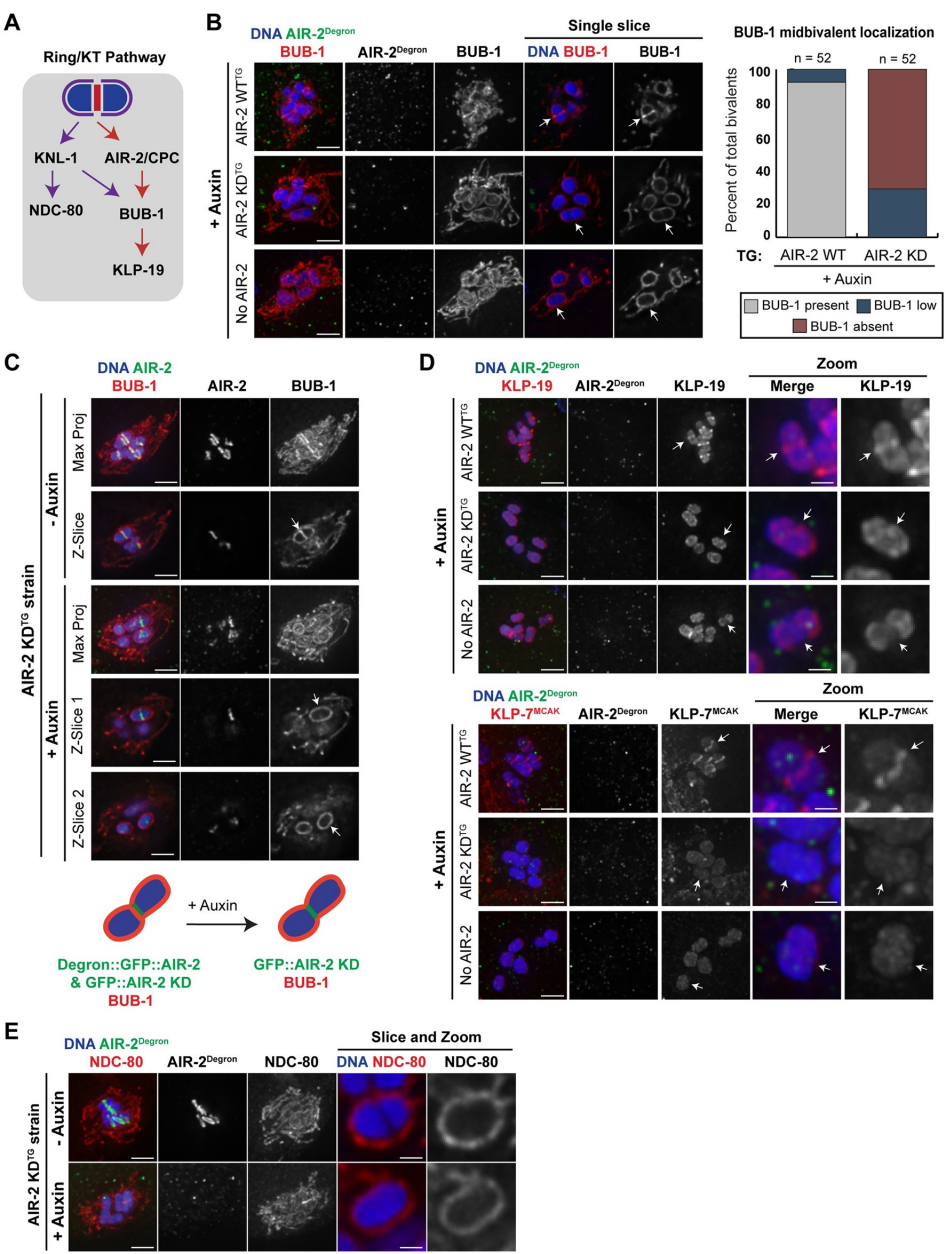
AIR-2 does not exclusively serve as a scaffold, and its kinase activity is required for ring complex assembly. (A) Schematic of known pathways of kinetochore (purple) and RC (red) assembly. (B) Localization of BUB-1 (red) on bivalents in the presence and absence of AIR-2 kinase activity; in all images endogenous AIR-2 (degron antibody, green) was depleted by auxin treatment. When wild-type transgenic AIR-2 is present in the oocyte, BUB-1 localizes to the midbivalent, cup-like kinetochores, and filaments. However, in the absence of AIR-2 or in the presence of kinase-dead AIR-2, BUB-1 does not localize to the midbivalent. The right two columns are single z-slices, to better show the midbivalent region (denoted with arrows). Quantification shown to the right of the images. (C) All images are of the AIR-2 KD^TG^ strain, in the absence (top) and presence (bottom) of auxin. Arrows indicate the midbivalent region. In the presence of WT AIR-2, BUB-1 (red) localizes to cup-like kinetochores surrounding the bivalents, filaments within the spindle, and to the midbivalent region. In the absence of AIR-2 kinase activity, BUB-1 does not load onto the RC even when kinase-dead AIR-2 (AIR-2 antibody, green) achieves midbivalent localization. Z-slice images highlight examples of chromosomes where kinase-dead AIR-2 is present but BUB-1 is not; schematic below images. (D) Localization of KLP-19 (red, top panel) and KLP-7^MCAK^ (red, bottom panel) on bivalents (blue) in the presence and absence of AIR-2 kinase activity; all images are in the presence of auxin. When wild-type transgenic AIR-2 is present (top row), KLP-19 and KLP-7^MCAK^ localize to the midbivalent. However, in the absence of AIR-2 or in the presence of kinase-dead AIR-2, these proteins are present on chromosomes but are not at the midbivalent. Arrows denote midbivalent region. (E) Localization of kinetochore component NDC-80 (red) on bivalents (blue); images are of the AIR-2 KD^TG^ strain in the presence and absence of auxin. Kinetochore morphology appears unaffected in the absence of AIR-2 kinase activity; zoomed images are of a single z-slice instead of a full projection, to more clearly show kinetochore organization. Bars = (A,C,D,E) 2.5μm; (E, zoom) = 0.85μm.

Upon nuclear envelope breakdown, the CPC facilitates the assembly of a structure called the ring complex (RC) around the central region of each bivalent. The RC is built in layers: the CPC forms an inner layer closest to the DNA, which in turn recruits outer layer components. AIR-2 is required for targeting the kinase BUB-1 to the RC, which is in turn required for targeting KLP-19, a plus-end-directed motor (Fig 3A) [14,15]. However, it has not been clear whether AIR-2 simply serves as a scaffold for RC assembly by recruiting other components through protein-protein interactions, or whether its kinase activity is also important. Our finding that kinase-dead AIR-2 is able to associate with most bivalents in the absence of endogenous AIR-2 enabled us to address this question (Fig 2B; although AIR-2 KD often fails to localize to the midbivalent, it is on chromosomes in most cases and could thus theoretically still serve as a scaffold).

First, we treated AIR-2 KD^TG^ worms with auxin and assessed BUB-1 localization; we also stained oocytes with an antibody that recognizes the degron tag, to confirm that endogenous AIR-2 was depleted. When kinase-dead AIR-2 was expressed, similar to the “no AIR-2” condition, BUB-1 failed to localize to the midbivalent in a majority of bivalents (Fig 3B and S4A Fig). To verify that the loss of BUB-1 wasn’t simply due to a failure of kinase-dead AIR-2 to localize to the midbivalent in the analyzed oocytes, we repeated this experiment and co-stained with BUB-1 and AIR-2 antibodies; the AIR-2 antibody recognizes transgenic AIR-2 and therefore can be used to establish its presence on bivalents. This analysis confirmed that BUB-1 is not able to localize to the midbivalent even in the presence of kinase-dead AIR-2 (Fig 3C, bottom two rows, and S4B Fig). Of all quantified bivalents where AIR-2 KD was present at the midbivalent or mispatterned, 95% (57/60) lacked BUB-1 at the midbivalent. These findings suggest that the kinase activity of AIR-2 is essential for RC assembly, which suggests that this protein does not serve solely as a scaffold. Further supporting this conclusion, we found that KLP-19, which is dependent on BUB-1 for targeting to the RC, failed to localize to the midbivalent in the absence of AIR-2 kinase activity (Fig 3D), even when the kinase-dead version of AIR-2 was present as a scaffold for RC assembly (S4C Fig).

In addition to its RC localization, BUB-1 localizes to kinetochores, which form cup-like structures around the ends of *C*. *elegans* bivalents, and to filaments within the spindle [32]. Although we found that AIR-2 kinase activity is required to target BUB-1 to the RC, its localization to the kinetochore and to filaments was unaffected in the absence of AIR-2 kinase activity (Fig 3B and 3C, S4A and S4B Fig). To determine if the localization of other kinetochore components was affected under these conditions, we stained for NDC-80, a known AIR-2 substrate that localizes to the kinetochore independently of BUB-1 [15] (Fig 3A). We found that NDC-80 also localizes to the kinetochore in the absence of AIR-2 kinase activity (Fig 3E), indicating that this activity is not required for targeting kinetochore components to chromosomes.

We also investigated whether AIR-2 kinase activity was required for the localization of KLP-7^MCAK^, another RC component. Previous reports have shown that KLP-7^MCAK^ localizes to both the RC and chromatin in oocytes [33–35] (Fig 3D), and can be phosphorylated by AIR-2 *in vitro* [34], but the requirements for targeting this protein to the RC have not been examined. In both the “no AIR-2” and the AIR-2 kinase-dead conditions, we found that the RC population of KLP-7^MCAK^ was absent, but the chromatin population was still present (Fig 3D), even when kinase-dead AIR-2 was present on the bivalent (S4C Fig). Together, these findings support the idea that AIR-2 kinase activity facilitates RC assembly but does not play a major role in targeting proteins to other regions of the bivalent.

### AIR-2/Aurora B kinase activity regulates the SUMO pathway in oocytes

We further investigated the mechanism by which AIR-2 kinase activity affects ring complex assembly. Recent reports have demonstrated that SUMOylation is important for RC assembly and stability [16,36]. Moreover, AIR-2, KLP-19, and BUB-1 can be SUMOylated *in vitro*, suggesting that SUMOylation of these components enables the targeting of other components with SUMO-interacting motifs to enable RC assembly [16,37]. Therefore, we wanted to test whether AIR-2 kinase activity mediates ring complex assembly via RC SUMOylation.

**Fig 4:**
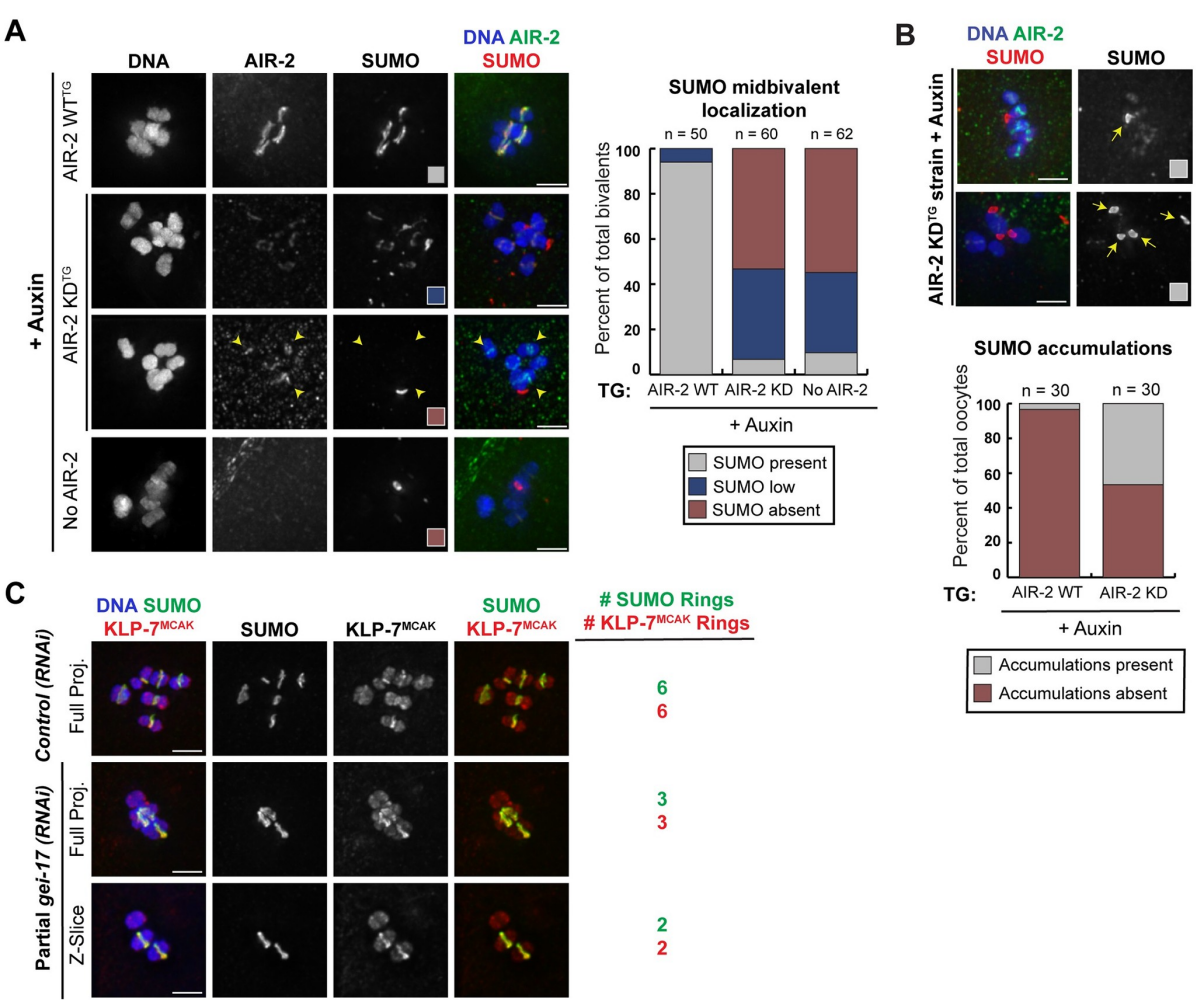
Aurora B kinase activity is required for SUMO-dependent ring complex assembly. (A) SUMO (red) in oocytes expressing transgenic wild-type AIR-2 (top row), kinase-dead AIR-2 (middle two rows) and no AIR-2 (bottom row); all images are in the presence of auxin to degrade endogenous AIR-2, so the AIR-2 antibody (green) denotes the localization of transgenic AIR-2. SUMO localizes to the midbivalent when wild-type transgenic AIR-2 is expressed, but in the presence of kinase-dead AIR-2, SUMO was only weakly associated with chromosomes or entirely absent; arrowheads show examples of chromosomes where kinase-dead AIR-2 was chromosome-associated, but SUMO was not present. In the no AIR-2 condition, SUMO was entirely absent from chromosomes. Note that the SUMO signals in the bottom two rows appear chromosome-associated in the full projection images, but are not in the same focal planes as the chromosomes and therefore are likely to be SUMO accumulations, analogous to those shown in panel B. Quantification to the right of the images. (B) In the absence of AIR-2 kinase activity, many oocytes displayed accumulations of SUMO (red, highlighted with arrows) that did not appear to be associated with the chromosomes. Quantification shown below the images. (C) KLP-7^MCAK^ localization to the RC is dependent on SUMOylation. In control oocytes, all six RCs are SUMOylated (green) and KLP-7^MCAK^ (red) localizes to all six bivalents (blue). After 36 hours of *gei-17(RNAi)* (partial depletion), only 3 RCs were SUMOylated and KLP-7^MCAK^ localized only to those 3 RCs; bottom image shows a single z-slice to highlight KLP-7^MCAK^ localization more clearly. Bars = 2.5μm.

We found that SUMO failed to localize to the midbivalent when endogenous AIR-2 was degraded with auxin (“No AIR-2”) (Fig 4A), consistent with the previous finding that the CPC is required for SUMO localization [16]. Notably, although RC SUMOylation was restored in the strain containing the wild-type AIR-2 transgene, SUMO showed reduced or undetectable localization to RCs when only kinase-dead AIR-2 was expressed. Since the failure of SUMO targeting could in some cases be caused by a failure of kinase-dead AIR-2 to target to bivalents (Fig 2B; a fraction of oocytes lacked AIR-2 KD on chromosomes), we separately quantified bivalents that had kinase-dead AIR-2 localized, and found that SUMO was low or absent on 96.1% (98/102) (example in Fig 4A, arrowheads). Thus, the reduced RC SUMO staining is not solely due to defective targeting of kinase-dead AIR-2, indicating that AIR-2 kinase activity promotes RC SUMOylation.

Consistent with this hypothesis, we also noticed that instead of localizing to the six rings, SUMO often formed aberrant accumulations when AIR-2 was absent or when the kinase-dead version was present; the number of these accumulations were highly variable between the oocytes. These SUMO accumulations were amorphously shaped and instead of localizing to the RC, they were often present in the general vicinity of the oocyte chromosomes but away from the midbivalent region, where the ring complex is located (Fig 4B, arrows). When we stained for SUMO substrates AIR-2, KLP-19, and BUB-1 (Fig 3) or other CPC components (S3 Fig), we did not observe analogous accumulations. Thus, we speculate that SUMO may not be conjugated to its normal substrates under these conditions, resulting in pools of unconjugated SUMO. Notably, these pools have not been previously reported following depletion of GEI-17, a SUMO E3 ligase required for RC SUMOylation [16,37], suggesting that they are not a general consequence of preventing SUMO from localizing to the RC. Therefore, inhibiting AIR-2 kinase activity interferes with the SUMOylation of RC components in a unique way, implicating AIR-2 in regulation of the SUMO pathway.

We also wanted to do the converse experiment and determine if SUMOylation affects AIR-2. It has already been established that preventing RC SUMOylation by depleting GEI-17 does not prevent AIR-2 midbivalent localization [16,36], but we wanted to determine if SUMOylation impacts AIR-2’s kinase activity. To test this, we used a strain in which SUMO (SMO-1) was degron-tagged; in this strain SUMO is absent on all chromosomes but AIR-2 is still localized to the midbivalent. We found that H3S10p was present on 100% of the bivalents that lacked SUMO (54/54), suggesting that lack of SUMOylation does not significantly alter the kinase activity of AIR-2 *in vivo* (S4D Fig).

Since SUMO is required for the recruitment of BUB-1 and KLP-19 to the RC [16], the failure of these proteins to properly localize in the AIR-2 kinase-dead strain (Fig 3B, 3C and 3D, and S4C Fig) could be a consequence of defective SUMOylation. To determine if this could also explain the reduced KLP-7^MCAK^ RC localization (Fig 3D), we next tested whether KLP-7^MCAK^ also requires the SUMO pathway for RC targeting. To this end, we partially depleted the SUMO E3 ligase GEI-17 using RNAi, a treatment that we previously showed generates a mixture of SUMOylated and un-SUMOylated RCs [36] (Fig 4C). We then assessed KLP-7^MCAK^ and found that this protein only localized to the SUMOylated RCs, suggesting that KLP-7^MCAK^ also requires SUMO for targeting (Fig 4C). Thus, we propose that AIR-2 kinase activity promotes RC formation by enabling proper RC SUMOylation, which in turn facilitates the recruitment of components to the complex.

### AIR-2/Aurora B kinase activity is required for acentriolar spindle bipolarity

**Fig 5:**
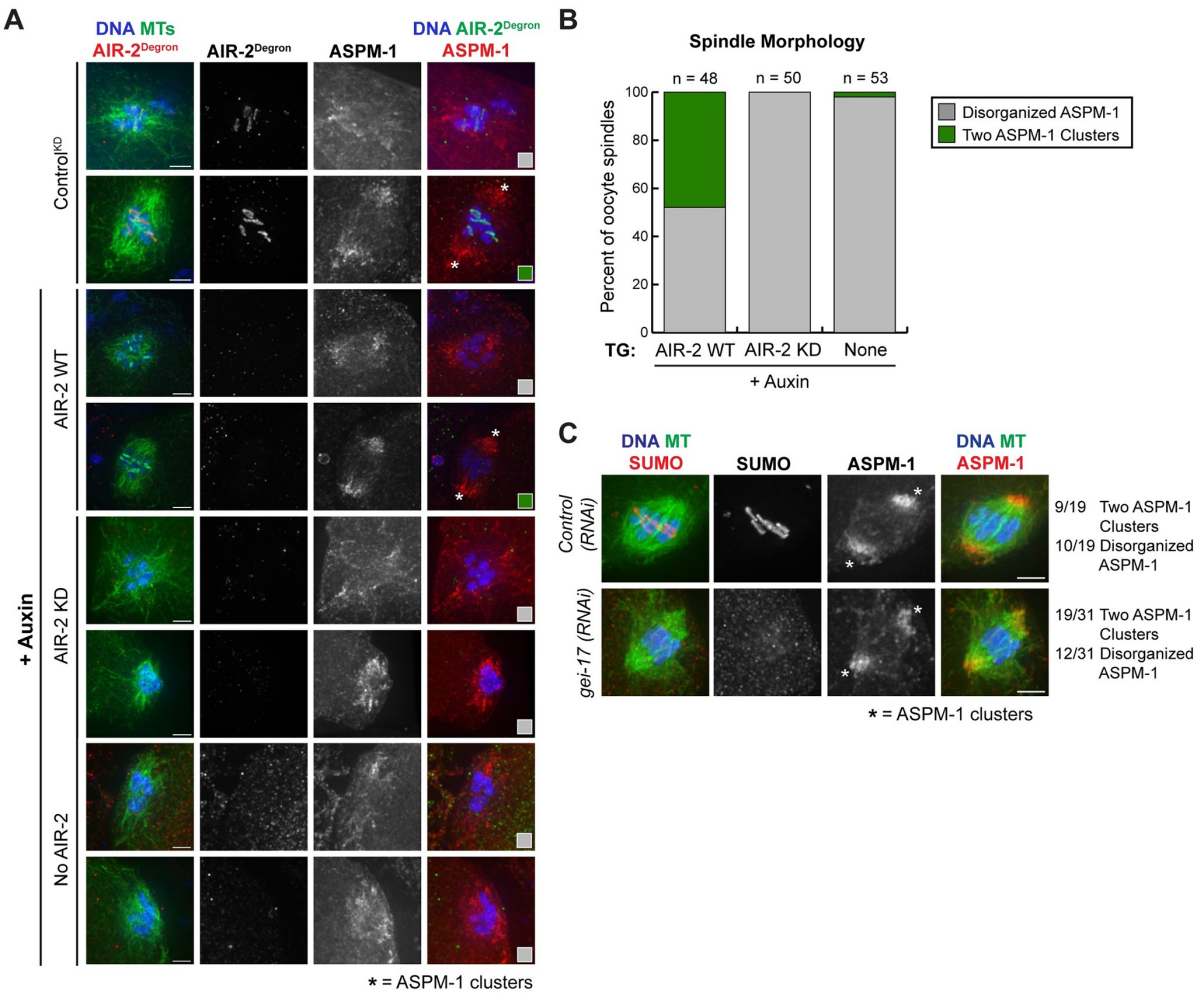
AIR-2 kinase activity is required for acentriolar spindle bipolarity. (A) Shown are microtubules (green), DNA (blue), and ASPM-1 (red, right column); ASPM-1 was used to mark the minus-ends of microtubules to assess spindle organization. The degron antibody (red, left column) was used to visualize endogenous AIR-2. In control worms expressing endogenous AIR-2 (the AIR-2 KD^TG^ strain without auxin; top two rows) or following depletion of endogenous AIR-2 in the presence of the wild-type AIR-2 transgene (+ auxin; rows 3 and 4), most spindles were either multipolar or bipolar, reflecting normal stages of spindle assembly. However, in the presence of only transgenic kinase-dead AIR-2 (rows 5 and 6) or with no AIR-2 (rows 7 and 8), spindles were either multipolar or had no distinct poles. Distinct ASPM-1 clusters that represent poles of bipolar spindles have been denoted by asterisks. (B) Quantification of spindle organization from the conditions shown in (A). Spindles were quantified as either “disorganized ASPM-1” to represent multipolar spindles and structures with no obvious poles (grey) or spindles containing two distinct ASPM-1 clusters to represent a bipolar spindle (green); examples of each category denoted by the colored boxes in part A. (C) SUMO (red, left column) and ASPM-1 (red, right column) localization following either control RNAi or partial *gei-17(RNAi)*. Depletion of GEI-17 prevents RC SUMOylation, but spindles are still bipolar (poles denoted with asterisks). Bars = 2.5μm.

Next, we wanted to determine if AIR-2 kinase activity is required for other meiotic events. Aurora B and other CPC components have been shown to promote spindle assembly in multiple organisms (reviewed in [1,38,39]) and there have been reports of disorganized spindles in *C*. *elegans* oocytes following depletion of CPC components [6,8,14,15]. However, a role for the CPC in spindle formation in this system has not been rigorously investigated, and what part of the assembly process is affected is unknown. Like most organisms, oocytes in *C*. *elegans* assemble acentriolar spindles. The stages of acentriolar spindle assembly in this system have been previously characterized. Following nucleation, microtubules are sorted into a structure with multiple poles marked by the microtubule minus-end-binding protein ASPM-1; these poles then coalesce to form a bipolar spindle [33,35,40,41]. To determine if AIR-2 kinase activity is required for this process, we assessed spindle morphology in our strains by imaging microtubules together with ASPM-1 and counting the number of ASPM-1 clusters to score spindle bipolarity (two clusters indicating a bipolar spindle); in order to focus on spindle assembly, any spindles that were clearly in anaphase (i.e., chromosomes segregating into distinct masses) were excluded. In the absence of auxin and in auxin-treated worms expressing the wild-type AIR-2 transgene, a substantial number of spindles were bipolar (Fig 5A and 5B). In contrast, in oocytes lacking AIR-2 or expressing kinase-dead AIR-2, there were almost no bipolar spindles and instead we largely observed disorganized ASPM-1, representing spindles with multiple ASPM-1-marked poles and also spindles that appeared disorganized and collapsed (Fig 5A and 5B). This result suggests that the kinase activity of AIR-2 is essential for spindle assembly; although microtubules nucleate and can form ASPM-1-marked poles without kinase activity, these poles fail to coalesce to establish bipolarity.

Since AIR-2 kinase activity is necessary for SUMO-dependent RC formation (Figs 3 and 4) and depletion of some individual RC proteins causes spindle defects [15,33–35], we next asked whether the spindle defects observed in the absence of AIR-2 kinase activity could be attributed to RC formation defects. To test this, we depleted the SUMO E3 ligase GEI-17 to prevent RC SUMOylation (Fig 5C). Under these conditions the RCs fail to form properly [16], but the kinase activity of AIR-2 is not directly perturbed. Notably, we found many examples of oocytes that lacked SUMO staining at the midbivalent where the spindles were still able to achieve bipolarity (Fig 5C and S5 Fig; also observed in [16]). Although some of these spindles appeared to have minor pole defects (i.e., splayed poles; Fig 5C and S5 Fig), they were organized along a single axis, in stark contrast to the kinase-dead AIR-2 conditions where bipolarity was almost never achieved (Fig 5A and 5B). Therefore, although the SUMO pathway and/or the RC may facilitate proper spindle assembly, our data suggest that the spindle defects observed in the absence of AIR-2 kinase activity cannot be solely explained by effects on the RC or SUMOylation. Thus, we propose that AIR-2 plays a role in spindle assembly that is distinct from its role in RC formation.

### AIR-2 kinase activity is essential for chromosome segregation in meiosis and mitosis

RNAi-mediated depletion of AIR-2 has been shown to prevent chromosome segregation in *C*. *elegans* oocytes [11,12], and there is evidence suggesting that AIR-2’s kinase activity is required for this process. Specifically, AIR-2 can phosphorylate the meiosis-specific cohesin subunit REC-8 *in vitro* [12] and it has been shown that REC-8 is phosphorylated at an AIR-2 consensus site, that phosphorylation of this site *in vivo* depends on AIR-2, and that mutating this site results in anaphase chromatin bridges, suggesting that AIR-2 phosphorylates REC-8 to mediate cohesin release [42]. Thus, we sought to test the prediction that AIR-2’s kinase activity is required for chromosome segregation.

**Fig 6:**
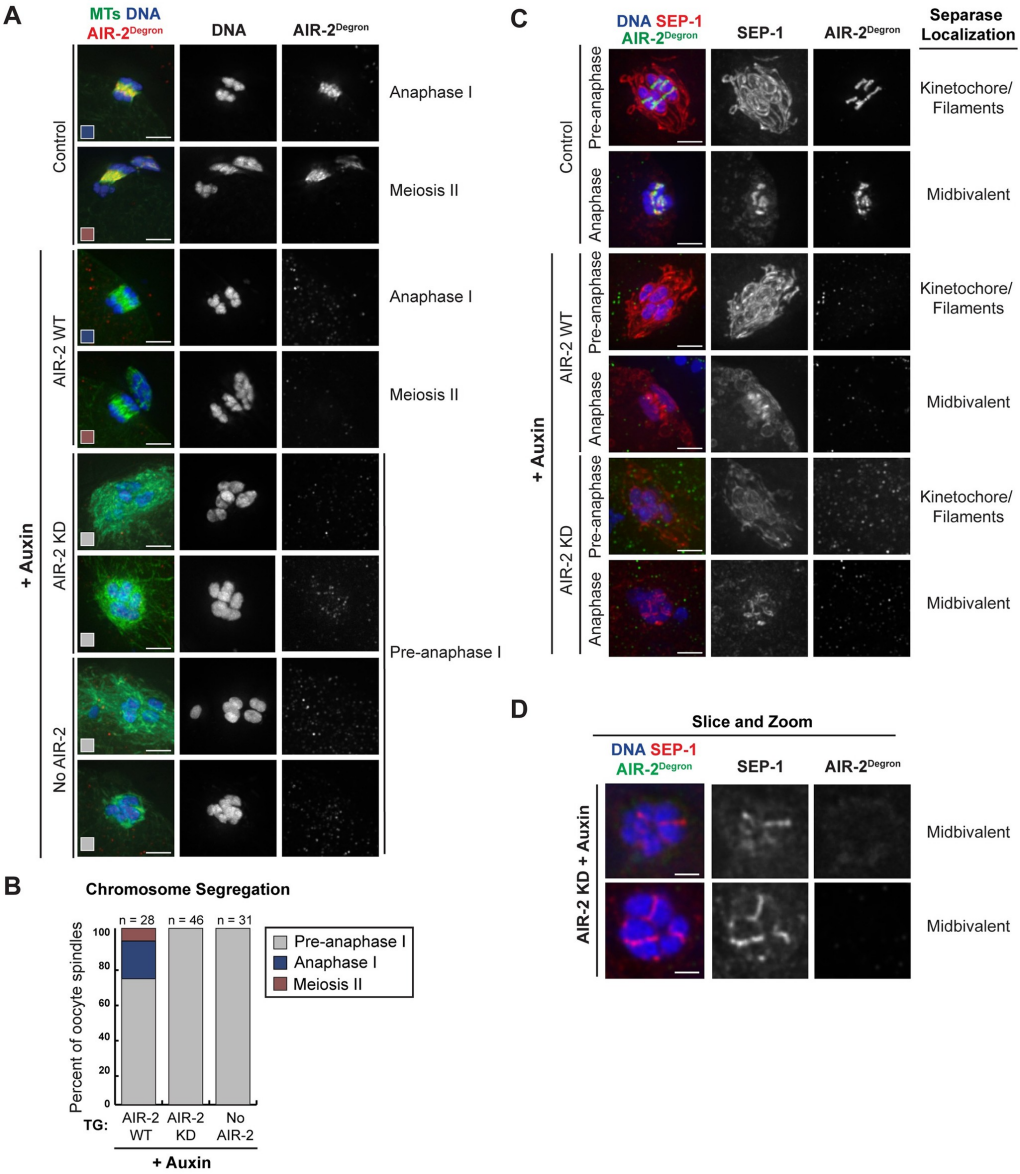
AIR-2 kinase activity is essential for chromosome segregation in oocytes. (A) Analysis of chromosome segregation in control worms without auxin treatment (top 2 rows), and in auxin-treated worms with either transgenic WT or KD AIR-2, or with no transgene (“No AIR-2”); endogenous AIR-2 was detected using the anti-degron antibody (red). Also shown are microtubules (green) and DNA (blue). While we observed Anaphase I and II chromosome segregation in control worms and in the presence of wild-type AIR-2, chromosome segregation failed to occur under the kinase-dead AIR-2 and no AIR-2 conditions. Colored boxes in the bottom of the images correspond to the graph in part B. (B) Quantification of chromosome segregation (for details see Materials and methods). (C) Shown are separase (red) and the degron antibody (green) to visualize endogenous AIR-2. In control oocytes, separase localizes to the kinetochore and filaments prior to anaphase but relocalizes to the midbivalent after anaphase onset [13] (top two rows); this same pattern was observed with auxin in the presence of the wild-type AIR-2 transgene (rows 3–4). When only kinase-dead AIR-2 was expressed, separase still relocalized to the midbivalent but there was no evidence of chromosome segregation (bottom 2 rows). (D) Single z-slices, highlighting examples of separase localization to the midbivalent in the absence of active AIR-2. Bars = (A,C) 2.5μm; (D) 0.85μm.

For this analysis, we included all oocyte spindles in our quantification, rather than excluding anaphase spindles as we did before; each spindle was classified as either “Pre-anaphase” (oocytes consisting of six intact bi-lobed bivalents, which could be either prometaphase or metaphase), “Anaphase I” (oocytes consisting of MI chromosomes segregating into two masses), or “Meiosis II” (oocytes with a polar body or with 12 separated homologous chromosomes, including both prometaphase, metaphase, and anaphase MII spindles). As expected, in untreated worms and in auxin-treated worms expressing the wild-type AIR-2 transgene, we observed all three categories (Fig 6A and 6B). Moreover, consistent with previous studies [11,12], the “Anaphase I” and “Meiosis II” categories were absent when we depleted endogenous AIR-2, indicating that segregation was completely blocked (Fig 6A and 6B). Notably, we obtained the same result when only kinase-dead AIR-2 was present (Fig 6A and 6B); we never observed cases where bivalents were able to separate into individual homologs, confirming the prediction that AIR-2’s kinase activity is essential for chromosome segregation.

For chromosomes to segregate in Anaphase I, cohesin must be degraded along the short-arm axis of the bivalent (i.e., the midbivalent region) [12]. Separase, which localizes to kinetochores and kinetochore filaments in prometaphase/metaphase [43], transitions to this region at anaphase onset [13,44] (Fig 6C, Control), presumably to cleave cohesin and enable chromosome separation; note that at this stage the separase-marked kinetochore filaments also disappear [32]. Given that we did not observe chromosome segregation in the AIR-2 kinase-dead condition, we wanted to determine if separase could target to the midbivalent region. Interestingly, we observed oocytes with separase at the midbivalent when only kinase-dead AIR-2 was expressed, suggesting that the segregation defects are unlikely to be the result of defects in separase targeting (Fig 6C and 6D). Additionally, this result suggests that the oocytes are not blocked in metaphase in the absence of AIR-2 kinase activity, since separase re-localization, which normally happens at anaphase onset, still occurred in the kinase dead condition (we scored oocytes with no kinetochore filaments, indicating that they were in anaphase, and 17/20 had separase at the midbivalent). Finally, these results also suggest that separase targeting to the midbivalent is not dependent on the presence of a fully formed ring complex.

We next assessed how defects caused by inhibiting AIR-2 kinase activity affected the 1-cell stage embryo. Consistent with the lack of meiotic chromosome segregation in the kinase-dead transgene condition, 100% of 1-cell stage embryos lacked polar bodies and contained excess DNA (S6A and S6B Fig). Moreover, similar to meiosis, there was limited evidence of chromosome segregation during mitosis (S6A Fig). Finally, we found that the kinase activity of AIR-2 was required for it to localize to mitotic chromosomes; while transgenic wild-type AIR-2 was able to localize to kinetochores at the 1-cell stage in the absence of endogenous AIR-2, the kinase-dead version was not (S6C Fig). Our work thus suggests that the kinase activity of AIR-2 promotes a series of essential events during meiosis and mitosis, a view supported by the fact that we observed 100% embryonic lethality when only kinase-dead AIR-2 was expressed (S6D Fig).

## Discussion

### New insights into Aurora B function

**Fig 7:**
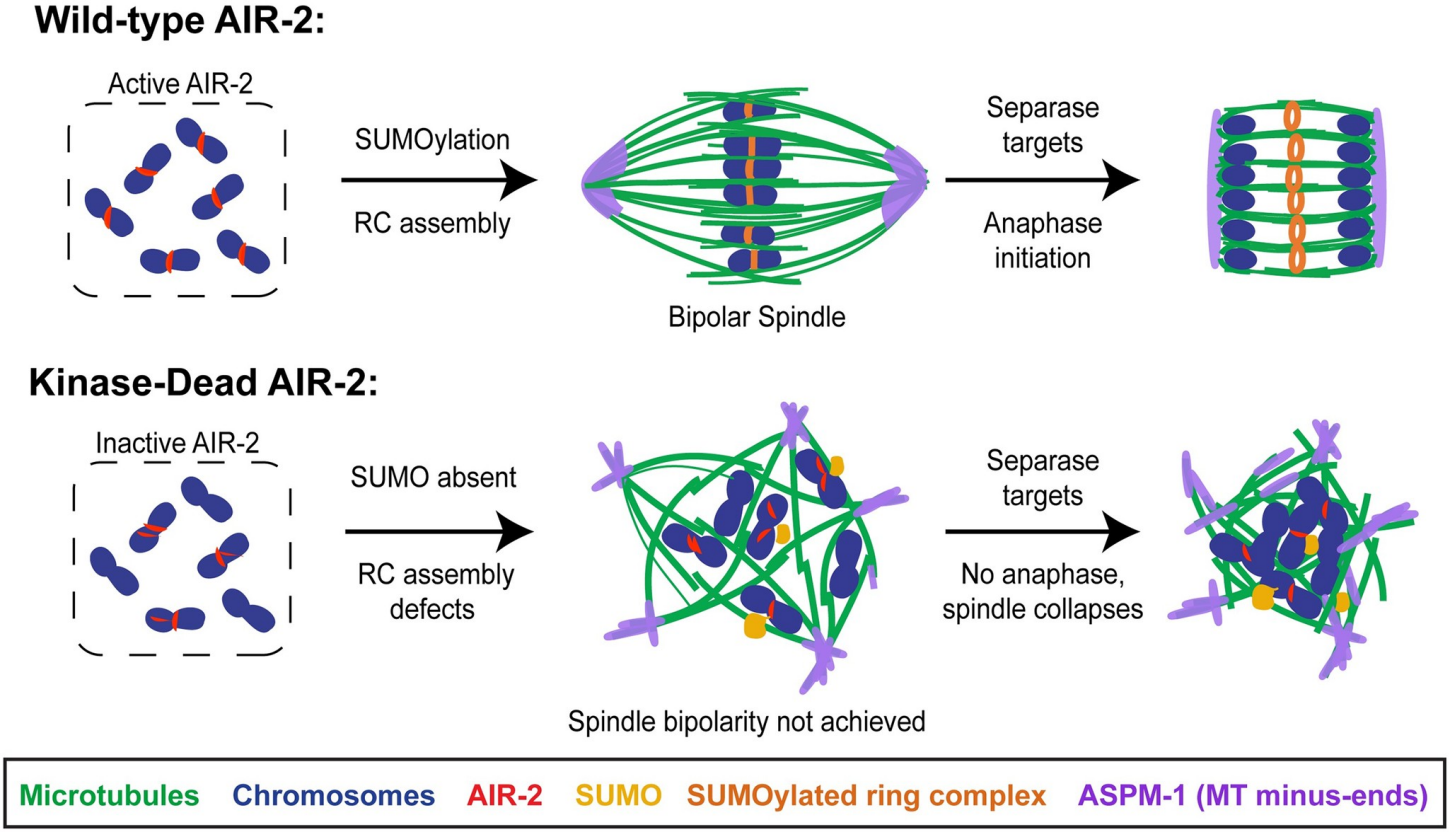
Model. Model depicting spindle assembly and chromosome segregation in wild-type oocytes (top) compared to oocytes lacking AIR-2 kinase activity (bottom). In wild-type oocytes, AIR-2 (red) localizes to the midbivalent, the RC forms and is SUMOylated (orange), and bipolar spindles form with ASPM-1 (purple) at the two poles. Chromosomes then separate and the RCs are left behind in the spindle midzone. In contrast, in the absence of kinase activity, AIR-2 is mispatterned on chromosomes, the RC is not SUMOylated, and SUMO (yellow) forms accumulations that are not chromosome-associated. Moreover, spindles do not achieve bipolarity and chromosome segregation fails.

In summary, we designed a degron-based approach that yielded new insights into Aurora B function in *C*. *elegans*. In wild-type oocytes, AIR-2 and other CPC components localize exclusively at the midbivalent and then downstream components are targeted to this region to enable RC assembly. Bipolar spindles then form and at anaphase onset, separase localizes to the midbivalent and chromosomes segregate (reviewed in [45]). In contrast, when endogenous AIR-2 was replaced by a kinase-dead version, we observed defects in all of these processes, demonstrating the importance of Aurora B’s kinase activity during cell division (Fig 7).

First, we found that AIR-2’s kinase activity is required for RC assembly. Kinase-dead AIR-2 either fails to localize to chromosomes or is mispatterned, and other CPC components also do not concentrate at the midbivalent. Moreover, even in conditions where the kinase-dead version localizes to chromosomes, downstream RC components fail to target. These RC formation defects appear to be due to effects on the SUMO pathway; instead of localizing to the RC, SUMO formed accumulations that were not chromosome-associated, suggesting that AIR-2 kinase activity promotes RC SUMOylation. These findings are noteworthy, since current models postulate that AIR-2 promotes RC assembly in ways that do not necessarily require kinase activity. First, since the RC is built in layers, with the CPC forming an inner layer closest to the DNA [15], it is thought that the CPC could serve as a scaffold for the recruitment of other proteins via protein-protein interactions. Additionally, since it has been proposed that SUMOylation of various RC components enables the recruitment of other proteins with SUMO-interacting motifs (SIMs), AIR-2 could recruit other RC components by acquiring a SUMO modification [16]. Although our findings are compatible with these models for RC assembly, we have now also revealed a requirement for kinase activity, demonstrating that AIR-2 likely regulates the assembly of this structure through phosphorylation as well.

We also found that in the absence of AIR-2’s kinase activity, spindles attempted to form but could not establish bipolarity and chromosomes failed to segregate during Anaphase I, despite the fact that separase was able to target to the midbivalent. Moreover, there were severe mitotic defects in the 1-cell stage embryo, with chromosome segregation errors and 100% embryonic lethality in conditions where kinase activity was absent. Furthermore, kinase-dead AIR-2 did not localize to mitotic kinetochores, suggesting that similar to meiosis, proper AIR-2 localization requires kinase activity. Thus, our work demonstrates that the kinase activity of AIR-2 is required for all of these crucial functions.

Although it is possible that some of the phenotypes that result from the loss of AIR-2 kinase activity are indirect (i.e., if an error in an early event is responsible for a later problem), there are published examples where defects in early events do not cause the same phenotypes we observe. For instance, AIR-2 and other CPC components are often mispatterned in the absence of AIR-2 kinase activity (Fig 1 and S3 Fig), so these chromosome structure defects could cause the failure to assemble the RC. However, bivalents containing multiple chiasmata have major CPC patterning defects but can still assemble RC components, demonstrating that proper chromosome structure is not a pre-requisite for RC assembly [17]. Similarly, it is possible that failure to assemble the RC in the AIR-2 KD^TG^ strain causes the later spindle assembly and chromosome segregation defects. However, preventing RC assembly by other means does not result in those phenotypes [16,36,37] (Fig 5C), making this less likely. Finally, the spindle defects that result from inhibition of AIR-2 could impact chromosome segregation. However, many groups have identified mutant conditions where spindle formation is aberrant, yet homologous chromosomes can still come apart in Anaphase I (a few examples from our own work are [13,41,44]), which we think makes this interpretation unlikely. Thus, we favor the idea that AIR-2’s kinase activity directly facilitates multiple processes.

### Potential AIR-2 targets in oocytes

An important area of future research will be to identify the phosphorylation targets of AIR-2 that mediate its proper localization and various functions. Since we noticed disrupted levels of AIR-2 at the midbivalent, one possibility is that similar to mitosis, AIR-2 regulates its own localization, where the kinase activity triggers a positive feedback loop to enable the protein to robustly accumulate at the midbivalent [46]. On the other hand, since there is evidence in other systems that CPC components can dimerize [47,48] it is also possible that loss of kinase activity could prevent this dimerization, as a result reducing AIR-2 localization to the bivalent. Previous studies have also proposed that Aurora B phosphorylates its binding partner INCENP as well as the T-loop of its own kinase domain; this latter phosphorylation is required to fully activate Aurora B [39]. These phosphorylation events may facilitate the localization of AIR-2 to the midbivalent and/or enable it to recruit downstream RC components. One way this could happen is if AIR-2 promoted the formation of the CPC by phosphorylating INCENP and/or itself; under this scenario kinase-dead AIR-2 would not be able to assemble into the CPC. If this were the case, this could be one reason why kinase-dead AIR-2 cannot serve as a scaffold for RC assembly; perhaps the whole CPC needs to be present to create the proper platform to recruit downstream RC components.

However, regardless of whether AIR-2 phosphorylates INCENP or itself, it is likely that AIR-2 also phosphorylates other targets to facilitate RC assembly. Notably, our studies suggest that AIR-2 promotes RC formation by regulating the SUMOylation of RC components; in the absence of AIR-2 kinase activity, SUMO accumulated away from the chromosomes and RC assembly failed. Although these SUMO accumulations may contain other proteins, they did not colocalize with any of the RC components we examined, raising the possibility that they may consist of SUMO that failed to conjugate to its substrates. Interestingly, knocking down the SUMO E3 ligase GEI-17 or the SUMO protease ULP-1 also results in RC SUMOylation defects but in those conditions SUMO was not reported to accumulate in the same manner [16,36]. Therefore, our findings suggest crosstalk between phosphorylation and SUMOylation and raise the possibility that AIR-2 may phosphorylate one or more components of the SUMO pathway.

In the absence of Aurora B kinase activity, we also observed defects in spindle assembly that could not be attributed entirely to defects in SUMOylation and RC formation. Prior work has shown that AIR-2 phosphorylates MCAK *in vitro* and that knockdown of MCAK leads to spindles with unfocused poles and long microtubules in *C*. *elegans* oocytes [33–35]. However, while spindles failed to achieve bipolarity in the AIR-2 kinase-dead conditions, we did not observe obvious excess microtubule growth, suggesting that AIR-2 might target additional spindle assembly factors. Another potential AIR-2 substrate that may affect the oocyte spindle is the kinetochore protein NDC-80. Previous experiments have shown that human NDC80 is an *in vitro* substrate of Aurora B [49], and in *C*. *elegans*, NDC-80 has been linked to controlling spindle pole number in oocytes [33]. In mouse oocytes, NDC80 depletion is also known to affect spindle organization [50]. Therefore, even though we found that NDC-80 localizes normally to the kinetochore, it is possible that its activity is altered in the absence of AIR-2 kinase activity. In the future it would be interesting to determine if non-RC associated pools of AIR-2 can phosphorylate kinetochore or spindle-associated factors to ensure spindle bipolarity.

Finally, we found that AIR-2 kinase activity is essential for chromosome segregation. Previous studies have shown that meiotic chromosomes fail to segregate after AIR-2 depletion [11,12], and we observed the same phenotype in the absence of AIR-2 kinase activity. One possibility we considered is that AIR-2 promotes chromosome segregation by regulating separase localization, since in mitotic cells, RNAi-mediated knockdown of Aurora B prevents the association of this protease with mitotic chromosomes [51]. However, we found that separase was still able to target to the midbivalent region at anaphase onset in the absence of AIR-2 kinase activity, so we infer that AIR-2 must be regulating meiotic chromosome segregation in other ways. Notably, this supports the conclusions of another study that provided evidence that AIR-2 phosphorylates the cohesin complex subunit REC-8 during meiosis to enable the release of sister chromatid cohesion [42]. However, the complete absence of chromosome segregation that we observed following inhibition of AIR-2 kinase activity is a more severe phenotype than that observed when the putative AIR-2 phosphorylation sites on REC-8 were mutated; in the latter case there were chromosome bridges but not a complete segregation failure. Therefore, in the future it will be important to identify other AIR-2 targets that could also promote chromosome segregation. In particular, it would be interesting to see if other cohesins such as COH-3/4 are also AIR-2 substrates, since these subunits also localize to midbivalent region and contain potential Aurora B kinase phosphorylation sites [52].

### Summary and future implications

Our work has uncovered multiple roles for AIR-2’s kinase activity during mitosis and oocyte meiosis and has revealed new functions for AIR-2 in the SUMO pathway and in promoting acentrosomal spindle bipolarity. This study therefore provides new insights into this important cell division regulator. In the future, our AIR-2 KD strain can be used to test whether any other documented functions of AIR-2 require kinase activity, for example in controlling other aspects of meiotic chromosome structure, such as condensin [20] and cohesin [12] patterning. Moreover, our degron-based approach should be widely useful in the future to probe the functions of other essential proteins. In *C*. *elegans*, full depletion of key cell division proteins using RNAi can cause developmental defects, whereas partial knockdown prevents a thorough analysis of protein function. Furthermore, although temperature-sensitive mutations can be powerful tools for conditional inactivation of proteins, such mutations can have unpredictable effects on protein function. A variety of evidence has also suggested that in *C*. *elegans*, mutations that cause strong temperature-sensitive defects in the embryo or at other developmental stages may not be as potently affected in the adult germ line. These limitations have complicated prior analysis of AIR-2 kinase activity. Our degron-based method allowed us to overcome these challenges, providing a means to differentiate kinase-dependent from independent roles. This work therefore lays the foundation for future studies that will further our understanding of the role of Aurora and other families of kinases during cell division.

## Materials and methods

### Strains

All crosses were conducted at 15°C. Genotypes were monitored by PCR using primers to detect the genes (or transgenes) of interest.

**CA1217**: *air-2(ie31[degron*∷*GFP*∷*air-2]); ieSi38[Psun-1*∷*TIR1*∷*mRuby*∷*sun-1 3’UTR*, *cb-unc-119(+)] IV*

**WH371**: *ojls50 [Ppie-1*∷*GFP*∷*air-2 + unc-119(+)]* [19]

**SMW20**: GFP∷AIR-2 WT^TG^ was generated by crossing strain CA1217 with strain WH371. *ojls50[Ppie-1*∷*GFP*∷*air-2 + unc-119 (+)]; air-2(ie31[degron*∷*GFP*∷*air-2]); ieSi38[Psun-1*∷*TIR1*∷*mRuby*∷*sun-1 3’UTR*, *cb-unc-119(+)] IV*

**JS533**: *[Ppie-1*∷*GFP*∷*air-2KD + unc-119(+)]*. This strain was made by cloning *air-2* into the pDONR201 vector, introducing a K65M mutation into the *air-2* gene, and then introducing it into *unc-119(ed3)* animals using bombardment [19].

**SMW17**: GFP∷AIR-2 KD^TG^ was generated by crossing strain CA1217 with strain JS533.

*[Ppie-1*∷*GFP*∷*air-2KD + unc-119(+)]*; *air-2(ie31[degron*∷*GFP*∷*air-2]); ieSi38[Psun-1*∷*TIR1*∷*mRuby*∷*sun-1 3’UTR*, *cb-unc-119(+)] IV*

**EU630**: *air-2(or207) I* (obtained from the CGC)

**PX3370**: *smo-1(syb3370[GFP*∷*degron*∷*smo-1]) I; unc119(ed3) III; ieSi38[Psun-1*∷*TIR1*∷*mRuby*∷*sun-1 3’UTR*, *cb-unc-119(+)] IV*

### Immunofluorescence

Immunofluorescence was performed as previously described [53]. Briefly, worms were picked into a drop of M9 buffer on poly-L-lysine slides and then cut to release oocytes. Slides were frozen in liquid nitrogen for 5–10 minutes, and then the coverslip was quickly removed with a razor blade. Embryos were fixed for 40–45 minutes in -20°C methanol, rehydrated in PBS, and blocked in AbDil (PBS plus 4% BSA, 0.1% Triton X-100, 0.02% Na-Azide). Primary antibodies were diluted in AbDil and incubated overnight at 4°C. Secondary antibodies were diluted in AbDil and incubated for 2 hours at room temperature. Hoechst 33342 (Invitrogen) was diluted 1:1000 in PBST (PBS + 0.1% Triton X-100) and incubated for 10–15 minutes at room temperature. Slides were washed with PBST between antibody incubations and mounted in 0.5% p-phenylenediamine in 90% glycerol, 20mM Tris, pH 8.8.

The following antibodies were used for immunofluorescence: rabbit anti-AIR-2 (1:1000; gift from Jill Schumacher), mouse anti-Degron (1:1000; MBL Life Sciences), pH3S10 (1:1000, Cell Signaling), rabbit anti-SEP-1 (1:200; gift from Andy Golden), mouse anti-α-tubulin-FITC (1:500; Sigma), mouse anti-SUMO (1:500, gift from Federico Pelisch), rabbit anti-CSC-1 (1:1000) [36], rabbit anti-BIR-1 (1:400, this study), rabbit anti-BUB-1 (1:2000) [36], rabbit anti-KLP-19 (1:2500, this study), rabbit anti-KLP-7^MCAK^ (1:300, this study). Alexa-fluor conjugated secondary antibodies (Invitrogen) were used at 1:500. BIR-1, KLP-19 and KLP-7^MCAK^ polyclonal antibodies were generated by Covance using recombinant GST-BIR-1 (Full length protein), GST-KLP-19 (amino acids 371–1084), and GST-KLP-7^MCAK^ (amino acids 1–249) as antigens (purification performed as in [36]). Antibody sera was then affinity purified and used at indicated concentrations.

### RNAi

From a feeding library [54,55], individual RNAi clones were picked and grown overnight at 37°C in LB with 100μg/ml ampicillin. Overnight cultures were spun down, plated on NGM (nematode growth media) plates containing 100μg/ml ampicillin and 1mM IPTG. Plates were dried overnight. Worm strains were synchronized by bleaching gravid adults and hatching overnight without food. Since full *gei-17* RNAi prevented RC SUMOylation, partial RNAi was performed. For these depletions, worms were grown until the L3-L4 stage on regular NGM/OP50 plates and then transferred to the RNAi plate 48 hours before preparing for immunofluorescence.

### Auxin treatments

Auxin treatments were performed as detailed in Divekar et al. [26] using the protocol “Long term auxin mediated deletion on plates”; that publication also includes information about applying this technique more broadly. Briefly, NGM plates were poured with 1mM Auxin and spotted using OP50 bacteria. Synchronized L4 stage worms were washed from regular NGM food plates using M9 and spotted on the auxin plates. The worms were allowed to grow on the auxin plates for about 18 hours, maturing the worms into young adults, prior to dissection. This long-term depletion protocol was chosen to ensure maximal depletion of endogenous AIR-2, while not affecting the development of the germ line (which is already formed at the L4 stage).

### Temperature shift experiments

For EU630 *(air-2(or207ts))* experiments, worms were shifted to 25°C at the L4 stage and then incubated until adulthood before dissection.

### Microscopy

All imaging was performed on a DeltaVision Core deconvolution microscope with a 100x objective (NA = 1.4) (Applied Precision). This microscope is housed in the Northwestern Biological Imaging Facility supported by the NU Office for Research. Slides were imaged at room temperature and image stacks were obtained at 0.2μm z-steps and deconvolved (ratio method, 15 cycles) using SoftWoRx (Applied Precision). All images in this study were displayed as full maximum intensity projections of data stacks encompassing the entire spindle structure unless otherwise noted.

### Embryonic lethality

Single L4 worms were placed on auxin plates and allowed to lay eggs for 24 hours at 15°C before being moved to another fresh auxin plate. The eggs were allowed to hatch for 24 hours and then the progeny (eggs and hatched worms) were counted. For each parent worm this process was repeated twice, resulting in three days of progeny being counted. For each condition, the progeny of at least 12 worms were scored.

### Western blotting

100 adult worms from auxin treatment or control plates were picked onto empty NGM plates, washed, and spun down in cold M9 twice. The M9 was removed to a final volume of approximately 20μl, then 20μl of 2x SDS sample buffer was added to the worms, and the sample was boiled at 95°C for 10 minutes. The 40μl sample was run in a single lane. Western blots were probed with rabbit anti-AIR-2 (1:5000), and mouse anti-tubulin (1:5000) as the loading control. ECL detection was used for Western blots.

### Ethanol fixation

Worms were either allowed to grow on regular NGM plates or transferred onto auxin plates overnight. Then about 10 worms were picked into a 5μl M9 drop on a microscope slide and the M9 was wicked away using Whatman paper. Then 5μl of 100% ethanol was added to the slide and allowed to evaporate, this process was repeated two more times. The worms were then mounted using a solution of 50% diluted Hoechst (1:1000 in M9) and 50% mounting media (0.5% p-phenylenediamine in 90% glycerol, 20mM Tris, pH 8.8). The slides were imaged within 5 hours of preparation.

### Image analysis and quantification

All images were quantified using softWoRx or ImageJ software; the localization of midbivalent proteins/markers was scored using sum projections; the z-stacks for these partial projections were selected to ensure that all of the chromosomes were included in the projection.

For quantification of protein levels on the midbivalent, in cases where the analyzed protein only localizes to the midbivalent, we measured the ratio of the background pixel intensity to that of the protein on the bivalent. The ratio cutoff for presence was determined based on control images. For low versus absent, we then used ratio cutoffs for each protein based on whether the protein was detectable on the bivalent in our sum projections. Each protein used a different cutoff to account for the inherent variability of using antibodies and non-specific staining (these ratios are listed for each Figure, below). For each condition, we quantified bivalents from at least 15 oocytes to minimize bias.

For quantification of mislocalization on the bivalent, if the fluorescent signal was detectable on chromosomes but was not restricted to the midbivalent region, the protein was scored as “mispatterned”.

Fig 1D: For pH3S10, bivalent to background ratio of over 3.0 was defined as present, 1.5–3.0 was defined as low, and less than 1.5 was defined as absent. In the no AIR-2 transgene strain, in the absence of auxin, H3S10p was present on 100% of all quantified bivalents (n = 52). Upon auxin addition, H3S10p was low on 9.6% of bivalents (n = 5), and absent on 90.4% of all quantified bivalents (n = 47). In the AIR-2 WT transgene strain, in the absence of auxin, H3S10p was present on 100% of all quantified bivalents (n = 52). In the presence of auxin, H3S10p was still present on 100% of bivalents (n = 52). In the AIR-2 KD transgene strain, in the absence of auxin, H3S10p was present on 100% of all quantified bivalents (n = 52). In the presence of auxin, H3S10p was low on 1.9% of bivalents (n = 1) and absent from 98.1% of all quantified bivalents (n = 51).

Fig 2B: For the AIR-2 transgene, bivalent to background ratio of over 2.0 was defined as present, 1.2–2.0 was defined as low, and 0.0–1.2 was defined as absent. In the AIR-2 WT condition, the AIR-2 transgene was present at the midbivalent in 90% of all quantified bivalents (n = 45), the transgene was present and mispatterned on 10% of all bivalents (n = 5). In the AIR-2 KD condition, the AIR-2 transgene was present at the midbivalent in 22.6% of all bivalents (n = 12). It was present but mispatterned on 20.8% of all quantified bivalents (n = 11), it was low and localized at the midbivalent in 26.4% of all bivalents (n = 14), and low and mispatterned on 11.3% of all bivalents (n = 6). Finally, the kinase-dead transgene was absent from 18.9% of all bivalents (n = 10).

Fig 2D: In the AIR-2 WT condition, CSC-1 was present on 86.8% of all quantified bivalents (n = 99), CSC-1 was mispatterned on 12.2% of all bivalents (n = 14) and absent from 0.88% of all bivalents (n = 1). In the AIR-2 KD condition, CSC-1 was present on 35% of all quantified bivalents (n = 63), was mispatterned on 63.3% of all bivalents (n = 114) and was absent from 1.7% of all bivalents (n = 3). In the no AIR-2 condition, CSC-1 was present on 40% of all quantified bivalents (n = 36), and was mispatterned on 60% of all bivalents (n = 54).

Fig 3B: Since BUB-1 localizes to kinetochores and filaments in addition to the midbivalent, to quantify BUB-1 we used a single z-slice at the middle of the bivalent, and drew a line scan using ImageJ across the longitudinal axis of each bivalent such that it would include the kinetochores as well as the midbivalent. We then noted the pixel intensity of the both the kinetochores and the midbivalent region of BUB-1. We then calculated the average pixel intensity of the two kinetochores (KT_avg_). Finally, we determined the ratio of the midbivalent BUB-1 intensity to that of the KT_avg_. A ratio of greater than 0.8 was defined as present, 0.35 to 0.8 was defined as low, and less than 0.35 was defined as absent. In conditions with AIR-2 WT, BUB-1 was present on 92.3% of all bivalents (n = 48), and low on 7.7% of all bivalents (n = 4). In the AIR-2 KD condition, BUB-1 was low on 28.8% of the bivalents (n = 15), and absent from 71.2% of all bivalents (n = 37).

Fig 4A: For SUMO levels, bivalent to background ratio of over 3.0 was defined as present, 1.5–3.0 was defined as low, and 0.0–1.5 was defined as absent. In the AIR-2 WT conditions, SUMO was present on 94% of bivalents (n = 47), and was low on 6% of bivalents (n = 3). In the AIR-2 KD condition, SUMO was present on 6.7% of bivalents (n = 4), was low on 40% of bivalents (n = 24), and was absent from 53.3% of all bivalents (n = 32). In the no AIR-2 condition, SUMO was present on 9.7% of bivalents (n = 6), was low on 35.5% of bivalents (n = 22), and was absent from 54.8% of all bivalents (n = 34).

Fig 4B: In the AIR-2 WT condition, SUMO accumulations were present in 3.3% of all quantified oocytes (n = 1) and were absent in 96.7% of oocytes (n = 29). In AIR-2 KD condition, SUMO accumulations were present in 46.7% of all quantified oocytes (n = 14) and were absent in 53.3% of oocytes (n = 16).

Fig 5B: Spindle morphology was quantified as “disorganized ASPM-1” to represent multipolar and collapsed spindles or “two distinct ASPM-1 clusters” to represent bipolar spindles. In the AIR-2 WT condition, 52.1% of the spindles had disorganized ASPM-1 (n = 25) and 47.9% of the spindles had two distinct ASPM-1 clusters (n = 23). In the AIR-2 KD condition, 100% of the spindles had disorganized ASPM-1. In the no AIR-2 condition, 98.1% of the spindles had disorganized ASPM-1 (n = 52) and 1.9% of the spindles had two distinct ASPM-1 clusters (n = 1).

Fig 6B: Stages were determined by assessing the presence/absence of polar bodies, by determining whether the spindles contained bivalents or individual homologs, and by looking for cases of active chromosome segregation (i.e. two separate masses of DNA that were on an anaphase spindle); the sizes of the DNA bodies were used to distinguish bivalents from individual homologs. Spindles with six bivalents and no polar bodies were defined as “Pre-anaphase I”. Spindles with two segregating masses of chromosomes and no polar bodies were defined as “Anaphase I”. Spindles with six individual homologs with one polar body and spindles with two segregating masses of chromosomes with one polar body were grouped into the “Meiosis II” category. Examples of each of these stages are shown in Fig 6A. Of the 28 quantified spindles in the AIR-2 WT condition, 71.4% were in Pre-anaphase I (n = 20), 21.4% were in Anaphase I (n = 6), and 7.2% were in Meiosis II (n = 2). Of the 46 and 31 spindles quantified in the AIR-2 KD and no AIR-2 condition, respectively, all of the spindles were in Pre-anaphase I, which means that there was no evidence of Meiosis I chromosome segregation.

S2B Fig: Western blot films were scanned in TIFF format and quantified using Photoshop. For pixel intensity measurements, the blots were first inverted. The background intensity was subtracted for each condition and then the measurement was obtained as a percentage of the background subtracted loading control (tubulin) for the given condition. The same sized area was used for all the measurements. For the band representing endogenously-expressed degron∷GFP∷AIR-2 (top band, 66kD), the values (represented as a % of the corresponding tubulin control band) are 22.1 (GFP∷AIR-2 WT^TG^ strain minus auxin), 8.1 (GFP∷AIR-2 WT^TG^ strain plus auxin), 23.0 (GFP∷AIR-2 KD^TG^ strain minus auxin) and 5.0 (GFP∷AIR-2 KD^TG^ strain plus auxin). These values reflect a decrease in endogenously-expressed degron-tagged AIR-2 upon auxin addition, and also show that the level of the endogenously-expressed AIR-2 is similar in the two strains. For the band representing transgenic GFP∷AIR-2 (bottom band, 61kDa), the values are 36.9 (GFP∷AIR-2 WT^TG^ strain minus auxin), 51.5 (GFP∷AIR-2 WT^TG^ strain plus auxin), 26.0 (GFP∷AIR-2 KD^TG^ strain minus auxin) and 42.3 (GFP∷AIR-2 KD^TG^ strain plus auxin). Comparison of these values in the two strains suggests that the transgenic WT AIR-2 is expressed at higher levels than transgenic KD AIR-2, and both of these transgenes appear to be expressed at higher levels than endogenously-expressed AIR-2. This analysis also suggests that the expression of transgenic AIR-2 may increase when endogenously-expressed AIR-2 is depleted using auxin, though a more rigorous analysis would be needed to verify that observation.

S2C Fig: For image quantification, images were deconvolved, and a sum projection was made from 15 slices encompassing all of the bivalents in the oocyte. A 47 pixel ellipse ROI was drawn around an area encompassing the bivalents using ImageJ, and the GFP∷AIR-2 intensity of that area was recorded. The final GFP intensity values were then calculated by subtracting a 47 pixel ellipse ROI of background GFP∷AIR-2 intensity from that recorded for the area encompassing the bivalents. The data is depicted in the scatter box plot, with the lines corresponding to the minimum, quartile 1, median, quartile 3, and maximum.

S3D Fig: In the AIR-2 WT condition, BIR-1 was present on 91.6% of all quantified bivalents (n = 33), and mispatterned on 8.4% of bivalents (n = 3). In the AIR-2 KD condition, BIR-1 was present on 26.6% of all quantified bivalents (n = 8), and mispatterned on 73.4% of bivalents (n = 22). Similarly, in the AIR-2 WT condition, ICP-1 was present on 100% of all quantified bivalents. In the AIR-2 KD condition, ICP-1 was present on 27% of all quantified bivalents (n = 13), and mispatterned on 73% of bivalents (n = 35).

S6B Fig: In the AIR-2 WT condition, 3.3% of all quantified 1-cell embryos (n = 1) had no polar bodies, 10% had 1 polar body (n = 3), and 86.7% had 2 polar bodies (n = 26). In the AIR-2 KD and no AIR-2 conditions, 100% of all quantified 1-cell embryos had no polar bodies.

S6D Fig: In the AIR-2 WT condition, 4.4% of all progeny from 12 worms were inviable in the presence of auxin. In the AIR-2 KD and no AIR-2 conditions, 100% of the progeny from 15 worms, were inviable in the presence of auxin.

## Supporting information

S1 Fig The *air-2(or207)* mutant does not display meiotic defects. (A) *air-2(or207)* mutants have substantial H3S10 phosphorylation (red) at both the permissive temperature of 15°C and the restrictive temperature of 25°C, demonstrating that this mutant is not fully kinase-dead. Also shown are microtubules (green, left column) and DNA (blue), to show the spindle, and SUMO (green, right column) to show that the RC also assembles at both temperatures. (B) *air-2(or207)* mutants do not display defects in RC assembly at either 15°C or 25°C, as AIR-2 (green, right column), and SUMO (red) still concentrate at the midbivalent. (C) *air-2(or207)* mutants do not display defects in spindle assembly at either 15°C or 25°C, as bipolar spindles form with ASPM-1 (red) at the two poles. Bars = 2.5μm.
(TIF)

S2 Fig Levels of degron-tagged AIR-2 and kinase-dead AIR-2 transgenes in worm strains. (A) Table detailing the versions of AIR-2 protein that are expressed and degraded in each of the generated strains in the presence and absence of auxin. (B) Western blots using an anti-AIR-2 antibody of whole worm samples of the GFP∷AIR-2 KD^TG^ and GFP∷AIR-2 WT^TG^ transgenic strains in the presence and absence of auxin, to evaluate the levels of degron∷GFP∷AIR-2 expressed from the endogenous locus (66 kDa) and GFP∷AIR-2 expressed from the bombarded transgenic constructs (61 kDa). Comparison of the endogenously-expressed bands shows a decrease upon auxin addition, though it is worth noting that these samples were generated from whole worms, while AIR-2 was only depleted in the germ line. The AIR-2 WT and AIR-2 KD transgenic constructs appear to be expressed at somewhat higher levels than endogenous AIR-2 (quantification in Materials and Methods). This is also quantified in part C in oocytes (rather than whole worm samples) using fluorescence intensity. (C) Quantification of absolute fluorescence intensity in oocytes expressing kinase-dead (GFP∷AIR-2 KD^TG^) and degron-GFP-tagged AIR-2 in the presence and absence of auxin. Worms were fixed with ethanol (which preserves GFP fluorescence) and then fluorescence levels were measured. Upon auxin addition, fluorescence decreases (due to degradation of endogenous degron∷GFP∷AIR-2). Note that the fluorescence level in the strain expressing transgenic GFP∷AIR-2 KD in the presence of auxin (reflecting the level of transgenic kinase-dead AIR-2) is higher than the fluorescence level of the endogenously-expressed degron∷GFP∷AIR-2, suggesting that the transgene may be overexpressed relative to endogenous AIR-2 in the germ lines of the generated strains. However, it is possible that if depletion of degron-GFP-tagged endogenous AIR-2 was incomplete, then this could also contribute to the fluorescence reading in the GFP∷AIR-2 KD strain. (D) Sample images and zooms of ethanol-fixed oocytes from intact worms that are quantified in part (C). Bars = 10μm; Zoom = 2.5μm.
(TIF)

S3 Fig CPC components are mispatterned in the absence of AIR-2 kinase activity. (A) Short-term auxin treatment (4 hours on auxin plates) resulted in depletion of endogenous AIR-2 (shown with the degron antibody, red), and GFP∷AIR-2 KD^TG^ (AIR-2 antibody, green) to be mislocalized or entirely absent from the midbivalent. Thus, short-term auxin depletion results in similar defects as long-term (overnight) auxin depletion. (B) CPC component BIR-1 (red) is mispatterned when only kinase-dead AIR-2 is expressed. Arrow indicates the location of the midbivalent, highlighting that the protein is not concentrated in that region in the absence of AIR-2 kinase activity. (C) CPC component ICP-1 (red) is mispatterned when only kinase-dead AIR-2 is expressed. Arrows indicate the location of the midbivalent, highlighting that the protein is not concentrated in that region in the absence of AIR-2 kinase activity. (D) Quantification of BIR-1 and ICP-1 on bivalents in the GFP∷AIR-2 KD^TG^ and GFP∷AIR-2 WT^TG^ transgenic strains (in the presence of auxin to degrade endogenous AIR-2), demonstrates that most bivalents have CPC patterning defects in the absence of AIR-2 kinase activity. Bars = 2.5μm; Zoom = 0.85μm.
(TIF)

S4 Fig Defects resulting from loss of AIR-2 kinase activity. (A) Shown are DNA (blue) and BUB-1 (red), which localizes to the kinetochore and the RC. In the absence of AIR-2 (bottom) or the presence of only kinase-dead AIR-2 (middle row), BUB-1 does not localize to the RC, and the midbivalent gap in the DNA staining is gone (arrowheads). (B) Shown are DNA (blue), BUB-1 (red), and GFP∷AIR-2 KD^TG^ (visualized with an AIR-2 antibody, green). Supporting the data shown in Fig 3C, these additional examples show that in the presence of auxin, GFP∷AIR-2 KD^TG^ can be patterned at the midbivalent or mispatterned. However, the presence of this kinase-dead version of AIR-2 is not sufficient to recruit downstream RC protein BUB-1, suggesting that AIR-2 does not merely act as a scaffold for ring complex assembly. (C) Zoom of bivalent with KLP-19 or KLP-7^MCAK^ (red), and GFP∷AIR-2 KD^TG^ (visualized with an AIR-2 antibody, green). The images show that in cases where the kinase-dead version of AIR-2 localizes to the midbivalent, it cannot recruit the downstream proteins KLP-19 and KLP-7^MCAK^. (D) Shown are DNA (blue), pH3S10 (red), and SUMO (green) in oocytes where RCs are SUMOylated (Control) and where RCs are unSUMOylated (in a strain where SMO-1 is degron-tagged; GFP∷Degron∷SUMO). In both conditions, pH3S10 persists on the bivalents. Bars (A, C) = 0.85μm (B, D) = 2.5μm.
(TIF)

S5 Fig GEI-17 depletion does not prevent spindle bipolarity, but does lead to mild spindle pole defects. Oocyte spindles formed following partial depletion of GEI-17 to prevent RC assembly. Bipolar spindles form, but spindle poles (marked by ASPM-1, red) are often partially split or splayed (arrows). Note that this ASPM-1 antibody occasionally shows non-specific staining on the chromosomes (e.g. the image on top row) [14]. Bar = 2.5μm.
(TIF)

S6 Fig Lack of AIR-2 kinase activity results in mitotic defects. (A) Shown are microtubules (green), DNA (blue) and endogenous AIR-2 (degron antibody, red) in 1-cell stage mitotic embryos. Chromosomes segregate and polar bodies (arrows) are present in control worms and in worms expressing the wild-type AIR-2 transgene. In contrast, segregation fails when AIR-2 is absent or when only kinase-dead AIR-2 is expressed. (B) Quantification of polar bodies in 1-cell embryos. The “zero polar bodies” category reflects a lack of meiotic chromosome segregation in the absence of AIR-2 or in the presence of kinase-dead AIR-2, further supporting the analysis presented in Fig 6. (C) AIR-2 transgene localization (assessed using the AIR-2 antibody, red) in 1-cell stage embryos following the depletion of endogenous AIR-2 (visualized with the degron antibody, middle row). Transgenic wild-type AIR-2 localizes to mitotic chromosomes but kinase-dead AIR-2 does not. (D) Quantification of embryonic lethality after auxin treatment; n’s represent the number of full broods counted. 4.4% embryonic lethality was observed in the strain expressing the AIR-2 WT transgene, compared to 100% lethality in the absence of AIR-2 or in the presence of kinase-dead AIR-2. Bars = (A) 10μm; (C) 2.5μm.
(TIF)
