# Supplementary figures and images for: A degron-based strategy reveals new insights into Aurora B function in C. elegans
Source: PLoS Genet. 2021 May 20;17(5):e1009567. doi: 10.1371/journal.pgen.1009567 (PMC8172070; doi:10.1371/journal.pgen.1009567)

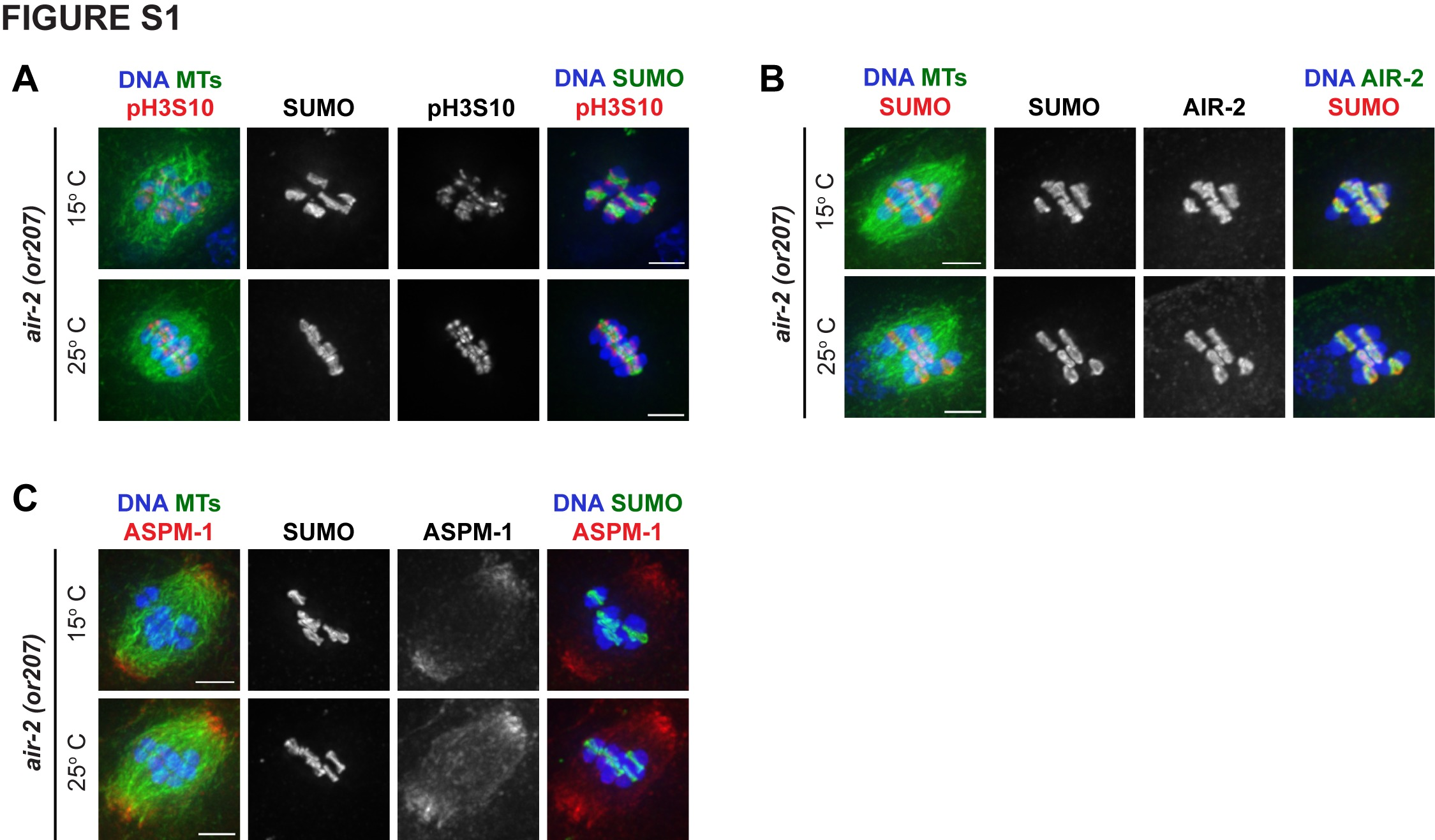

Supplement: S1 Fig — (A) air-2(or207) mutants have substantial H3S10 phosphorylation (red) at both the permissive temperature of 15°C and the restrictive temperature of 25°C, demonstrating that this mutant is not fully kinase-dead. Also shown are microtubules (green, left column) and DNA (blue), to show the spindle, and SUMO (green, right column) to show that the RC also assembles at both temperatures. (B) air-2(or207) mutants do not display defects in RC assembly at either 15°C or 25°C, as AIR-2 (green, right column), and SUMO (red) still concentrate at the midbivalent. (C) air-2(or207) mutants do not display defects in spindle assembly at either 15°C or 25°C, as bipolar spindles form with ASPM-1 (red) at the two poles. Bars = 2.5μm. (TIF) [file pgen.1009567.s001.tif]

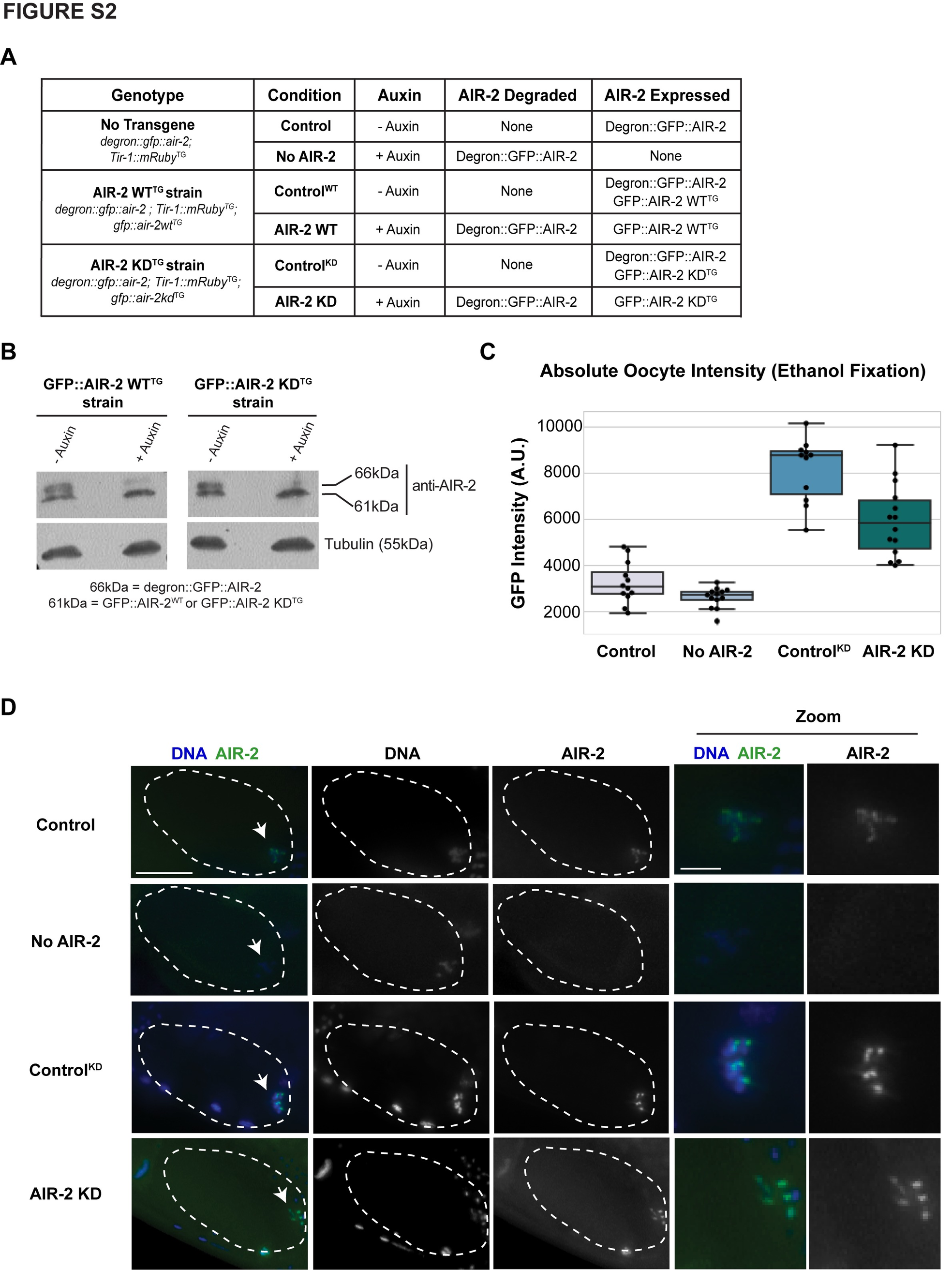

Supplement: S2 Fig — (A) Table detailing the versions of AIR-2 protein that are expressed and degraded in each of the generated strains in the presence and absence of auxin. (B) Western blots using an anti-AIR-2 antibody of whole worm samples of the GFP∷AIR-2 KDTG and GFP∷AIR-2 WTTG transgenic strains in the presence and absence of auxin, to evaluate the levels of degron∷GFP∷AIR-2 expressed from the endogenous locus (66 kDa) and GFP∷AIR-2 expressed from the bombarded transgenic constructs (61 kDa). Comparison of the endogenously-expressed bands shows a decrease upon auxin addition, though it is worth noting that these samples were generated from whole worms, while AIR-2 was only depleted in the germ line. The AIR-2 WT and AIR-2 KD transgenic constructs appear to be expressed at somewhat higher levels than endogenous AIR-2 (quantification in Materials and Methods). This is also quantified in part C in oocytes (rather than whole worm samples) using fluorescence intensity. (C) Quantification of absolute fluorescence intensity in oocytes expressing kinase-dead (GFP∷AIR-2 KDTG) and degron-GFP-tagged AIR-2 in the presence and absence of auxin. Worms were fixed with ethanol (which preserves GFP fluorescence) and then fluorescence levels were measured. Upon auxin addition, fluorescence decreases (due to degradation of endogenous degron∷GFP∷AIR-2). Note that the fluorescence level in the strain expressing transgenic GFP∷AIR-2 KD in the presence of auxin (reflecting the level of transgenic kinase-dead AIR-2) is higher than the fluorescence level of the endogenously-expressed degron∷GFP∷AIR-2, suggesting that the transgene may be overexpressed relative to endogenous AIR-2 in the germ lines of the generated strains. However, it is possible that if depletion of degron-GFP-tagged endogenous AIR-2 was incomplete, then this could also contribute to the fluorescence reading in the GFP∷AIR-2 KD strain. (D) Sample images and zooms of ethanol-fixed oocytes from intact worms that are quantified in part (C). [file pgen.1009567.s002.tif]

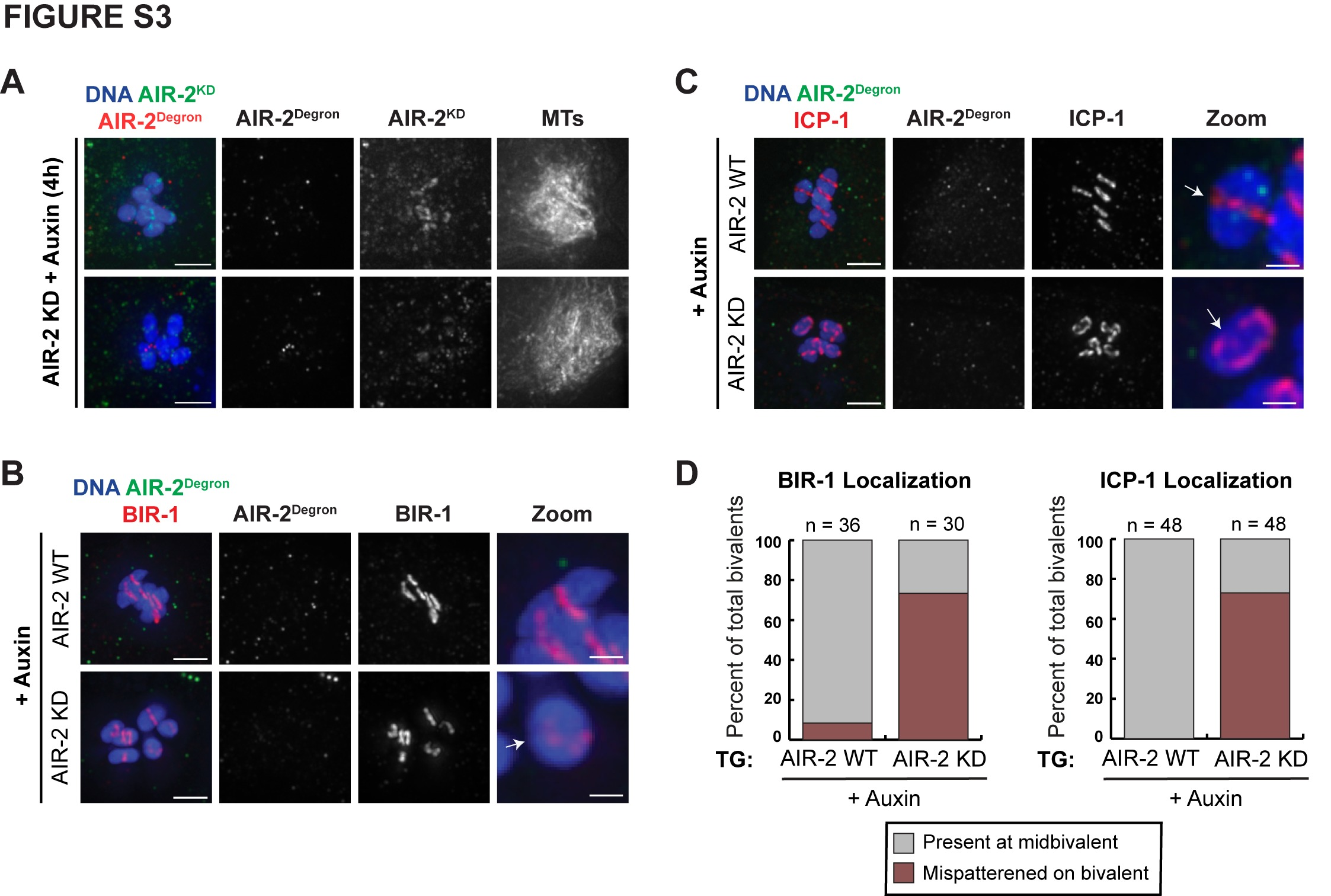

Supplement: S3 Fig — (A) Short-term auxin treatment (4 hours on auxin plates) resulted in depletion of endogenous AIR-2 (shown with the degron antibody, red), and GFP∷AIR-2 KDTG (AIR-2 antibody, green) to be mislocalized or entirely absent from the midbivalent. Thus, short-term auxin depletion results in similar defects as long-term (overnight) auxin depletion. (B) CPC component BIR-1 (red) is mispatterned when only kinase-dead AIR-2 is expressed. Arrow indicates the location of the midbivalent, highlighting that the protein is not concentrated in that region in the absence of AIR-2 kinase activity. (C) CPC component ICP-1 (red) is mispatterned when only kinase-dead AIR-2 is expressed. Arrows indicate the location of the midbivalent, highlighting that the protein is not concentrated in that region in the absence of AIR-2 kinase activity. (D) Quantification of BIR-1 and ICP-1 on bivalents in the GFP∷AIR-2 KDTG and GFP∷AIR-2 WTTG transgenic strains (in the presence of auxin to degrade endogenous AIR-2), demonstrates that most bivalents have CPC patterning defects in the absence of AIR-2 kinase activity. Bars = 2.5μm; Zoom = 0.85μm. (TIF) [file pgen.1009567.s003.tif]

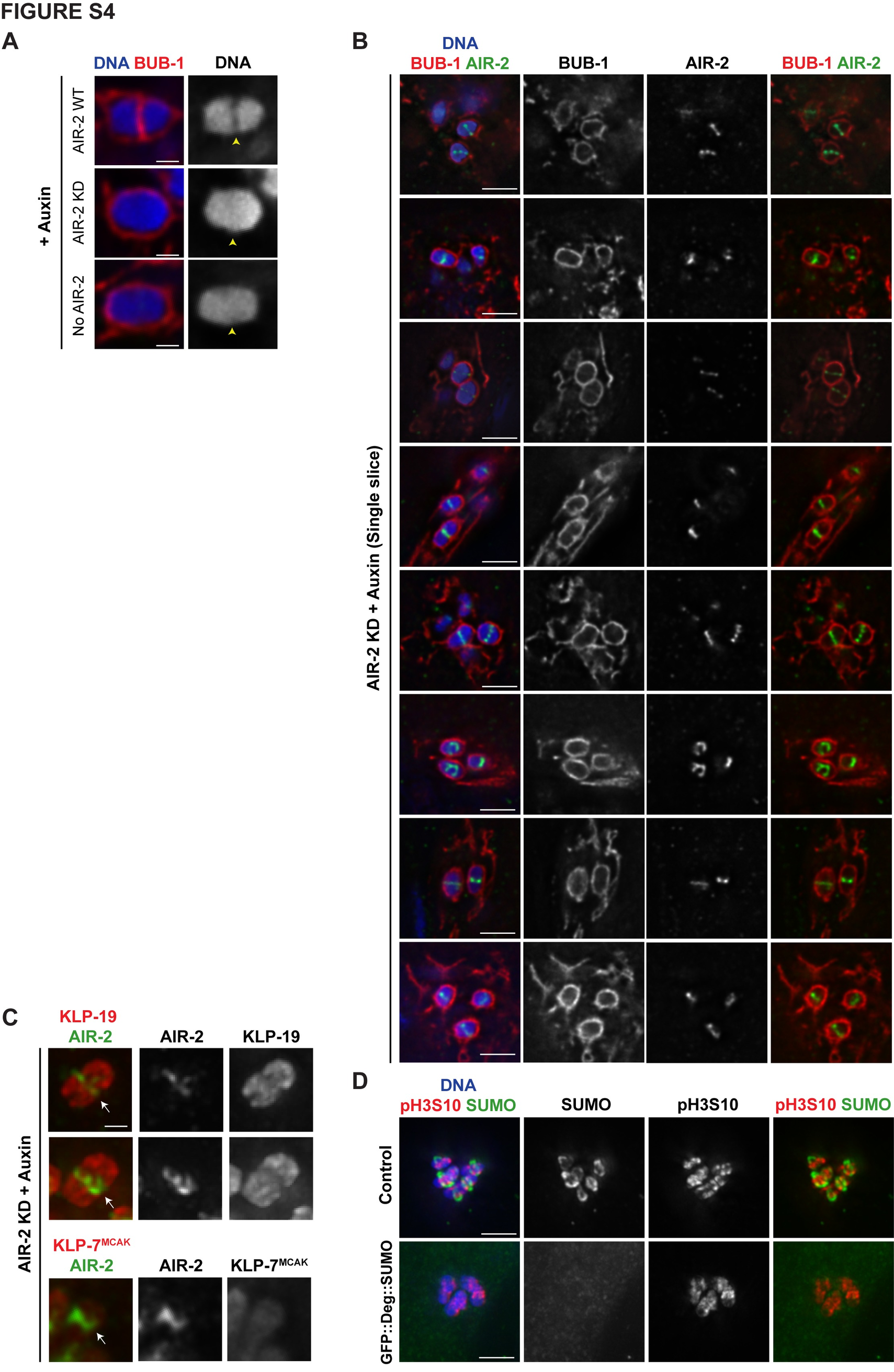

Supplement: S4 Fig — (A) Shown are DNA (blue) and BUB-1 (red), which localizes to the kinetochore and the RC. In the absence of AIR-2 (bottom) or the presence of only kinase-dead AIR-2 (middle row), BUB-1 does not localize to the RC, and the midbivalent gap in the DNA staining is gone (arrowheads). (B) Shown are DNA (blue), BUB-1 (red), and GFP∷AIR-2 KDTG (visualized with an AIR-2 antibody, green). Supporting the data shown in Fig 3C, these additional examples show that in the presence of auxin, GFP∷AIR-2 KDTG can be patterned at the midbivalent or mispatterned. However, the presence of this kinase-dead version of AIR-2 is not sufficient to recruit downstream RC protein BUB-1, suggesting that AIR-2 does not merely act as a scaffold for ring complex assembly. (C) Zoom of bivalent with KLP-19 or KLP-7MCAK (red), and GFP∷AIR-2 KDTG (visualized with an AIR-2 antibody, green). The images show that in cases where the kinase-dead version of AIR-2 localizes to the midbivalent, it cannot recruit the downstream proteins KLP-19 and KLP-7MCAK. (D) Shown are DNA (blue), pH3S10 (red), and SUMO (green) in oocytes where RCs are SUMOylated (Control) and where RCs are unSUMOylated (in a strain where SMO-1 is degron-tagged; GFP∷Degron∷SUMO). In both conditions, pH3S10 persists on the bivalents. Bars (A, C) = 0.85μm (B, D) = 2.5μm. (TIF) [file pgen.1009567.s004.tif]

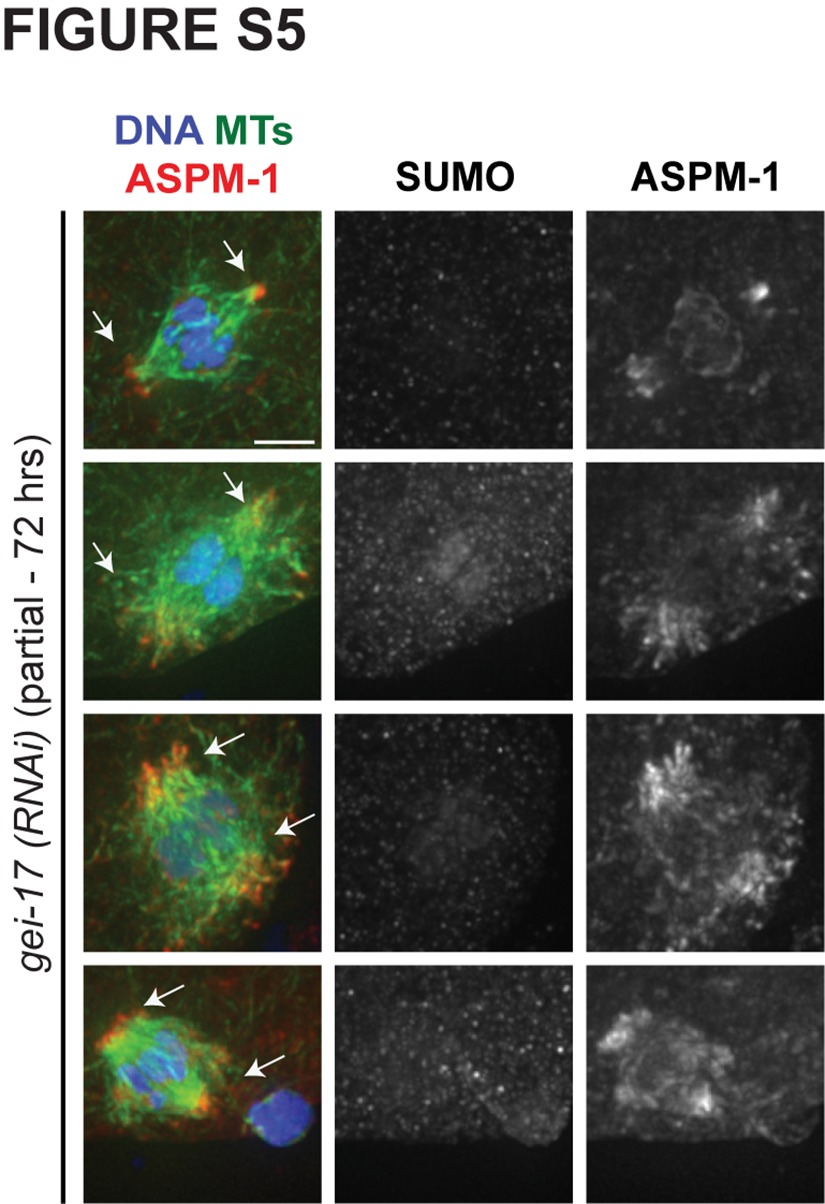

Supplement: S5 Fig — Oocyte spindles formed following partial depletion of GEI-17 to prevent RC assembly. Bipolar spindles form, but spindle poles (marked by ASPM-1, red) are often partially split or splayed (arrows). Note that this ASPM-1 antibody occasionally shows non-specific staining on the chromosomes (e.g. the image on top row) [14]. Bar = 2.5μm. (TIF) [file pgen.1009567.s005.tif]

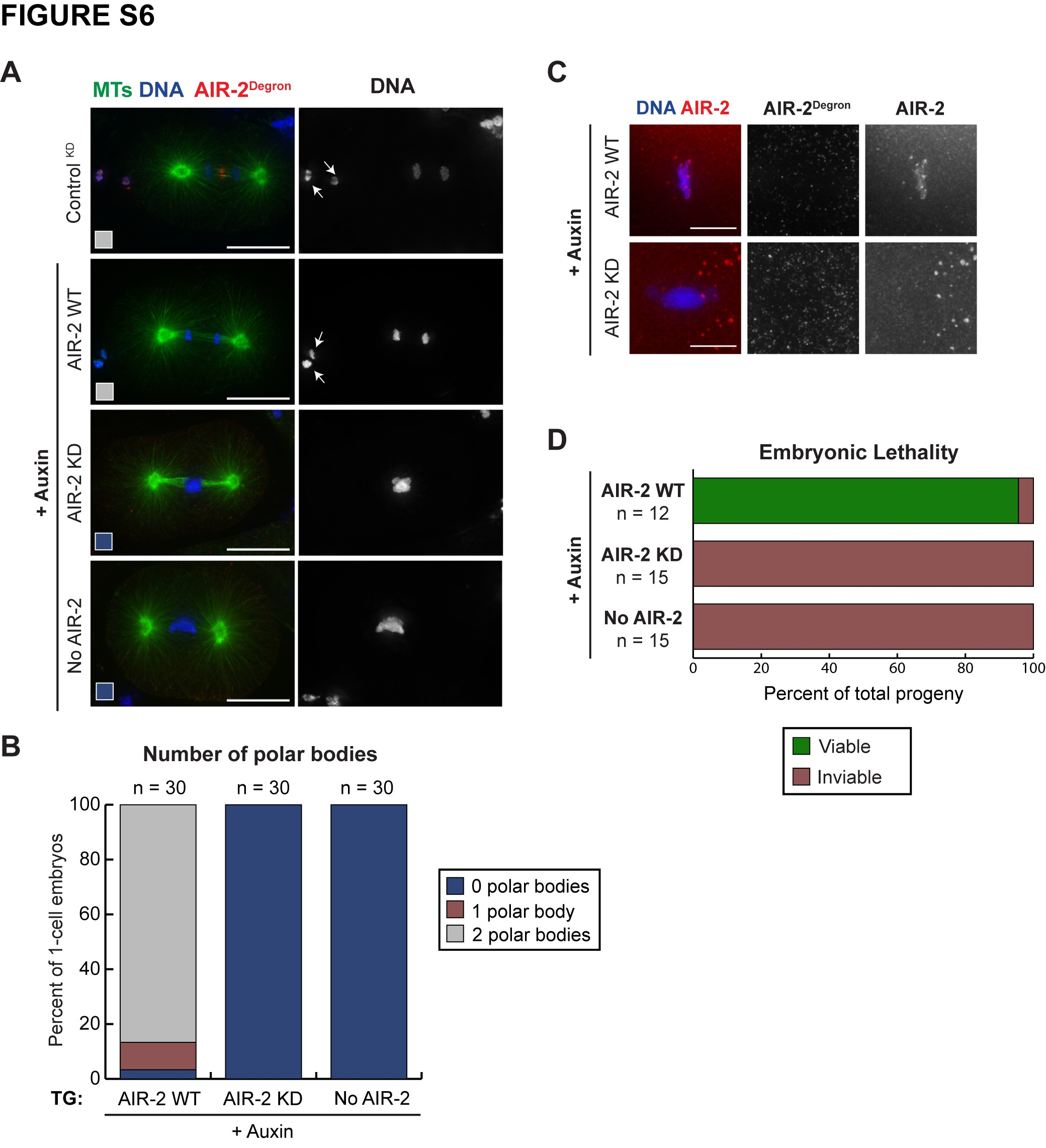

Supplement: S6 Fig — (A) Shown are microtubules (green), DNA (blue) and endogenous AIR-2 (degron antibody, red) in 1-cell stage mitotic embryos. Chromosomes segregate and polar bodies (arrows) are present in control worms and in worms expressing the wild-type AIR-2 transgene. In contrast, segregation fails when AIR-2 is absent or when only kinase-dead AIR-2 is expressed. (B) Quantification of polar bodies in 1-cell embryos. The “zero polar bodies” category reflects a lack of meiotic chromosome segregation in the absence of AIR-2 or in the presence of kinase-dead AIR-2, further supporting the analysis presented in Fig 6. (C) AIR-2 transgene localization (assessed using the AIR-2 antibody, red) in 1-cell stage embryos following the depletion of endogenous AIR-2 (visualized with the degron antibody, middle row). Transgenic wild-type AIR-2 localizes to mitotic chromosomes but kinase-dead AIR-2 does not. (D) Quantification of embryonic lethality after auxin treatment; n’s represent the number of full broods counted. 4.4% embryonic lethality was observed in the strain expressing the AIR-2 WT transgene, compared to 100% lethality in the absence of AIR-2 or in the presence of kinase-dead AIR-2. Bars = (A) 10μm; (C) 2.5μm. (TIF) [file pgen.1009567.s006.tif]
